# Gut microbiota-derived 5-HTP penetrates the host blood–brain barrier and ameliorates autism symptoms

**DOI:** 10.1016/j.apsb.2026.06.025

**Published:** 2026-06-16

**Authors:** Bei Li, Qianqian Yang, Min Li, Junqing He, Zhiqi Zhang, Xuchun Wan, Zihao Mo, Ruizhen Li, Shihan Li, Weirong Xu, Man Gu, Hanning Huang, Wei Li, Ziming Huang, Hongju Yang, Wei-Hua Chen, Zhi Liu

**Affiliations:** a Hubei Key Laboratory of Resource Utilization and Quality Control of Characteristic Crops, College of Life Science and Technology, Hubei Engineering University, Xiaogan 432000, China; b Key Laboratory of Molecular Biophysics of the Ministry of Education, Hubei Key Laboratory of Bioinformatics and Molecular Imaging, Department of Biotechnology, College of Life Science and Technology, Huazhong University of Science and Technology, Wuhan 430074, China; c Key Laboratory of Molecular Biophysics of the Ministry of Education, Hubei Key Laboratory of Bioinformatics and Molecular Imaging, Center for Artificial Intelligence Biology, Department of Bioinformatics and Systems Biology, College of Life Science and Technology, Huazhong University of Science and Technology, Wuhan 430074, China; d Department of Child Health, Maternal and Child Health Hospital of Hubei Province, Tongji Medical College, Huazhong University of Science and Technology, Wuhan 430070, China; e Wuhan Children's Hospital, Tongji Medical College, Huazhong University of Science and Technology, Wuhan 430010, China; f Division of Geriatric Gastroenterology, The First Afliated Hospital of Kunming Medical University, Kunming 650032, China; g Gaozhou People's Hospital, Gaozhou 525200, China; h School of Biological Science, Jining Medical University, Rizhao 272111, China

**Keywords:** Autism spectrum disorder, 5-HTP, Gut microbiota, Blood–brain barrier, Tryptophan metabolites, MAPK, ERK, RTK

## Abstract

Autism spectrum disorder (ASD), a highly prevalent neurodevelopmental condition, is increasingly recognized for its strong association with the intestinal microbiome. However, the development of gut microbiota-targeted therapies has been impeded by a limited understanding of the molecular mechanisms underlying interactions between commensal bacteria and the host nervous system. In this study, shotgun metagenomic sequencing and UPLC–MS/MS targeted metabolic analyses identified altered tryptophan metabolites in the gut microbiota of both human ASD patients and ASD mouse models. Notably, we demonstrate that commensal bacteria-derived 5-hydroxytryptophan (5-HTP), metabolite of tryptophan, ameliorates anxiety, stereotypical and repetitive behaviors, as well as social deficits in these mouse models. Furthermore, 5-HTP is capable of crossing the blood–brain barrier and inhibits the overexpression of receptor tyrosine kinase (RTK) ligands, thereby suppressing the downstream RTK/MAPK/ERK signaling cascade. This inhibition subsequently normalizes the excessive stabilization of dendritic spines in the hippocampus in MeCP2 mouse. Our research demonstrates that gut microbiota producing 5-HTP improves ASD symptoms in various ASD animal models, elucidates the molecular mechanisms between gut microbiota and the onset and treatment of ASD, and provides a promising therapeutic approach for ameliorating ASD through the expression of neuron-regulated small molecules by gut indigenous bacteria.

## Graphical abstract

5-HTP produced by gut microbes has improved autism-related symptoms in a variety of mouse models of autism. 5-HTP crosses the blood–brain barrier and directly affects the development of neurons. 5-HTP inhibits the overexpression of receptor tyrosine kinase (RTK) ligands, thereby inhibiting the downstream RTK receptor/MAPK/ERK signaling cascade, and subsequently normalizes the over-stabilization of dendritic spines in the hippocampus.

**Figure:**
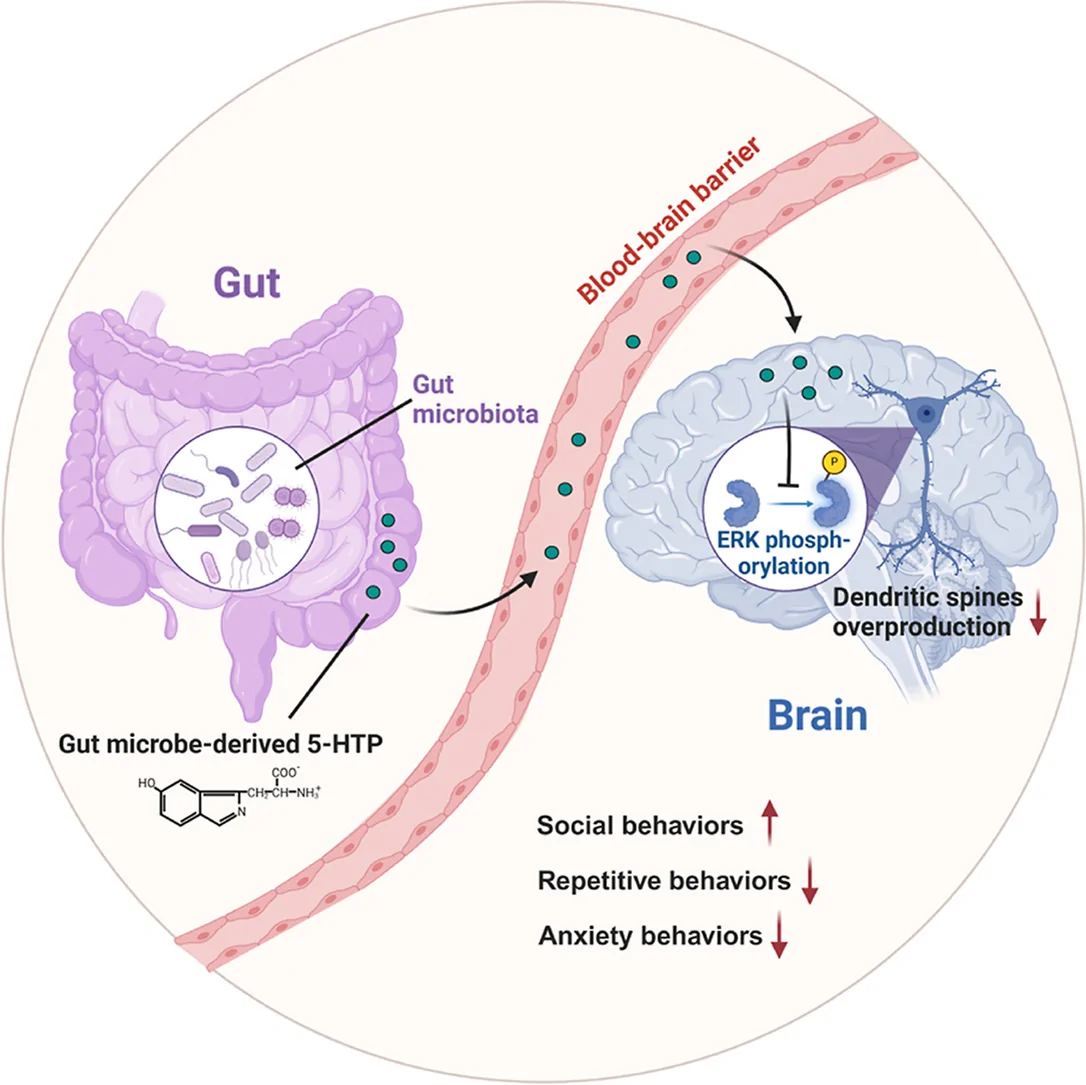

## 1 Introduction

Autism spectrum disorder (ASD) encompasses a group of neurodevelopmental conditions characterized by deficits in social communication and the presence of repetitive, stereotyped behaviors^1^. It is frequently accompanied by comorbidities such as anxiety disorders^2^ and epilepsy^3^. The incidence of ASD has risen sharply over recent decades, imposing significant financial and emotional burdens globally^4^, ^5^, ^6^, ^7^. Evidence suggests that ASD is highly heritable, with numerous risk genes identified, including MeCP2, SHANK3, Chd8, and SCN2A^8^, ^9^, ^10^, ^11^. Nonetheless, prenatal^12^^,^^13^, perinatal^14^, nutritional^15^^,^^16^, and environmental factors^17^ are also believed to contribute to ASD pathogenesis, and the precise etiology remains poorly understood.

Recent studies have demonstrated that the gut microbiota play a crucial role and serves as a therapeutic target for ASD. Children with ASD exhibit significant alterations in the microbiota composition^18^, ^19^, ^20^ and frequently suffer from gastrointestinal dysfunctions such as constipation, diarrhea, and abdominal pain^21^. ASD individuals with gastrointestinal (GI) symptoms tend to exhibit more severe autistic symptoms^22^ compared to those without GI symptoms. Accumulating evidence suggests that intestinal microbes can modulate GI and brain functions, subsequently impacting behaviors through the gut–brain axis. Notably, children with ASD show improvements in both ASD symptoms and GI symptoms following fecal microbiota transplantation (FMT) treatment^23^, ^24^, ^25^. Additionally, oral administration of specific bacterial taxa, such as *Lactobacillus plantarum*^26^, *Lactobacillus reuteri*^27^ and *Bifidobacterium infantis*^28^, has been shown to ameliorate the clinical symptoms in children with ASD.

Bidirectional communication between our central nervous system and gut microbiota, which is known as the “gut–brain axis”, has garnered significant attention in recent years. The underlying mechanisms by which gut microbiota ameliorate symptoms of ASD through the gut–brain axis are multifaceted and have been extensively investigated. Firstly, gut microbiota involves the restoration of compromised intestinal barrier function, thereby reducing the translocation of detrimental substances such as lipopolysaccharide (LPS) and subsequently mitigating systemic inflammation^29^. Secondly, gut microbiota exerts an influence on the host's social behavior patterns through the essential neural pathway of the vagus nerve, which plays a pivotal role in gut–brain communication^30^. Moreover, by modulating immune regulatory pathways, gut microbiota suppresses excessive production of pro-inflammatory cytokines in both the serum and brain, thus crucially impeding the development of autism-related behaviors^31^. Notably, metabolites derived from gut microbiota play a central role in the etiology and potential therapeutic avenues of ASD, serving as signaling molecules implicated in gut–brain communication and potentially directly modulating neuronal activity and synaptic plasticity within the brain^32^^,^^33^. For instance, the microbial metabolite *p*-Cresol has been shown to induce social behavior deficits by altering microbiota composition^34^. Treatment with propionate has been found to induce hyperactive, repetitive, and antisocial behaviors in rats^35^. Additionally, the gut microbe-derived metabolite 4-ethylphenyl sulfate (4EPS) has been observed to enter the brain, impair oligodendrocyte maturation, reduce myelination of neuronal axons, and induce anxiety-like behaviors in mice^36^. Conversely, the gut microbiota metabolite 5-aminovaleric acid (5AV) has been shown to reduce neuronal excitability in the prefrontal cortex and ameliorate ASD-like behaviors in BTBR T + tf/J (BTBR) mice^37^.

Recent studies have revealed that tryptophan metabolites are closely associated with gastrointestinal dysfunction and deficiencies in social interaction^20^^,^^38^^,^^39^. However, the molecular mechanisms linking these metabolites to ASD remain unclear. In this study, we identified a key tryptophan metabolite, 5-hydroxytryptophan (5-HTP), derived from gut microbes, and verified its function in both humanized and transgenic mouse models of autism. Our results show that gut microbiota-derived 5-HTP can penetrate the host blood–brain barrier (BBB) and is involved in the regulation of dendritic spine development. We propose a mechanism whereby metabolites produced by gut microbiota can directly interact with the host's nervous system *via* systemic circulation, thereby alleviating symptoms associated with autism. These findings provide novel pathophysiological insights and potential therapeutic targets for ASD and other neuropsychiatric disorders.

## 2 Materials and methods

### 2.1 Study subject recruitment

The study was approved by the Ethics Committee of Wuhan Children's Hospital, Tongji Medical College, Huazhong University of Science and Technology (Wuhan, China). All participants were recruited from the Hepatic Surgery Center of Wuhan Children's Hospital. An informed consent form was signed by each participant (or legal guardians) before enrollment. All participants underwent rigorous phenotyping using standardized protocols. The Diagnostic and Statistical Manual of Mental Disorders, 5th Edition (DSM-5) were utilized for the diagnosis of ASD children. Children with ASD (*n* = 20) met DSM-5 diagnostic criteria confirmed by both ABC (≥53) and CARS (≥30) scores. Exclusion criteria included: (1) neuroanatomical abnormalities (MRI/EEG-confirmed); (2) uncorrectable sensory impairments; (3) metabolic disorders and other chronic diseases; (4) hearing impairment; and (4) current psychotropic medication use (4-week washout). The Rome IV criteria for functional constipation were used for evaluating GI problems. The typically developed children (TD) as control were recruited from kindergartens. TD children with any pathological conditions, including GI problems were excluded. Children in ASD or TD group that are taking dietary supplements (including probiotics and prebiotics), antibiotics, or other medications in one month before the feces collection were excluded.

### 2.2 Extraction of fecal genomic DNA and whole-genome shotgun sequencing

Human fecal samples were collected from typically developing children and children with ASD. Parents collected and froze a single fecal sample from each subject. Frozen fecal samples were transported to lab immediately with a cold pack, and stored at −80 °C. Genomic DNA was isolated from approximately 100 mg of stool using the Magnetic Soil and Stool DNA Kit (TIANGEN, Beijing, China) according to the manufacturer's instructions. Extracted gDNA sheared by sonication into fragments and suitably sized fragments between 300 and 350 bp were selected using VAHTS DNA Clean Beads (Vazyme, Nanjing, China) to construct the DNA library. Ligated DNA was PCR amplified was purified by VAHTS DNA Clean Beads (Vazyme, Nanjing, China). Libraries were quantified using a Qubit 3.0 fluorometer (Thermo Fisher Scientific, Carlsbad, CA, USA), and sequenced on a DNBSEQ-T7 platform (MGI, Shenzhen, China) according to the manufacturers' protocol.

Quality filtering, trimming and de-multiplexing was carried out by a custom pipeline containing Trim Galore and cutadapt for adapter and quality trimming, and PRINSEQ for low-complexity filtering and sequence deduplication. In addition, Bowtie2 v2.2.1 was used to map reads to MetaPhlAn markers for the classification of bacterial species. Entries’ scores were summarized according to KO annotations. Each sample was scaled to 1M. KEGG Pathway analysis was conducted using EMPANADA.

### 2.3 Animal model

Specific pathogen-free (SPF) C57BL/6J mice (6 weeks old, male) used for FMT and microbe treatment were purchased from the Hubei Province Center for Disease Control and Prevention (Wuhan, China). The transgenic mouse model of ASD used in the present study has been previously described^40^^,^^41^. Mice with MeCP2 duplication on a pure FVB/N background were obtained from The Jackson Laboratory (MeCP2-TG, Stock No: 008679). MeCP2-TG mice were crossed to C57BL/6J mice to generate MeCP2-duplication mice and littermate controls. Only males were used for experiments. BTBR mouse model were obtained from Fenoch Biotechnology (Shanghai, China) Co., Ltd.

The sample size of 10–15 mice per group was determined based on the Chinese Society for Laboratory Animal Sciences guidelines (TICALAS 74-2019). All the mice were housed (no more than four per cage) under humidity- and temperature-controlled (22 ± 1 °C, 55 ± 5% humidity) conditions and a 12-h light/dark cycle with free access to food and water. For surgical procedures, anesthesia was induced and maintained with isoflurane (4% and 1.5%, respectively) delivered *via* precision vaporizer, and analgesia was provided using buprenorphine (0.1 mg/kg, s.c.) administered pre- and post-surgery. Euthanasia was performed by cervical dislocation under deep anesthesia (isoflurane overdose).

All procedures strictly conformed to the Guide for the Care and Use of Laboratory Animals published by the United States National Institutes of Health and were approved by the Institutional Animal Care and Use Committee (IACUC) of Huazhong University of Science and Technology (Approval No. [2020] IACUC-2996).

### 2.4 Fecal microbiota transplantation (FMT) and co-house of mice

For FMT, six-week-old C57BL/6J recipient mice received ABX cocktail treatment through drinking water, 0.2 g/L ampicillin (BBI, Shanghai, China), 0.1 g/L vancomycin, 0.2 g/L neomycin (BBI, Shanghai, China)and 0.2 g/L metronidazole (BBI, Shanghai, China), for two weeks, After the antibiotic treatment was completed, the drinking water of the mice was replaced with a 10% (*w*/*v*) polyethylene glycol (PEG, Aladdin, Shanghai, China) solution for 3 days to remove residual antibiotics and facilitate the transplantation of exogenous bacteria. Clinically collected fecal samples were weighed and added to sterile saline to 0.2 g/mL. It was vortex shaken and homogenized on ice. The supernatant was collected by centrifugation at 3000 rpm for 2 min at 4 °C. All these procedures were performed under anaerobic conditions. Then, each mouse was orally administered 200 μL of donor fecal supernatant by gavage once a day for 14 days.

The co-housing procedure was performed according to established methods^42^. Prior to initiating the co-housing experiment, all cages and feeding equipment were thoroughly sterilized and cleaned to eliminate potential microbial contamination. Newly weaned male FVB wild-type (WT) mice and FVB MeCP2-TG transgenic mice were co-housed at a 1:1 ratio in the same cage. Throughout the 4-week experimental period, all animals were provided with sterilized irradiated feed and clean, sterile drinking water. To enhance microbial exchange, we mixed bedding materials between groups at the start of co-housing and minimized cage changes to maintain stable microbial transfer conditions.

### 2.5 Behavior test

Before the behavioral tests, all mice were allowed to acclimate to the test room for at least 2 h prior to starting the test. SMART 3.0 video tracking software (Panlab Harvard, MA, USA) was used to record and evaluate the subject mice’s movements by independent blind observers.

Three-chamber test: The three-chamber social test was performed based on a published method^43^^,^^44^. The subject mouse was first habituated in an empty 102 cm × 47 cm × 45 cm plastic box divided into three equal-sized interconnected chambers. The mouse is placed in the central compartment of the box and allowed to explore freely for 10 min. Then, the mouse is placed the same chamber again and interact with an object in one side chamber or with a stranger mouse (stranger 1) in the other side chamber for another 10 min to test its sociability. Both of the object and the stranger mouse are in a same small cage. Next, this mouse is returned to the central compartment and assessed for social preference with the familiar mouse (stranger 1) relative to a novel mouse (stranger 2). The time spent sniffing or climbing on the cage containing either mouse or toy was measured to calculate the interaction time.

Marble burying (MB): The marbles burying test was modified from previously described protocol^45^. Subject mice were first habituated into cages filled with corn cob bedding containing 20 black glass marbles (1.5 cm diameter, four equidistant rows of five marbles each, 5 cm deep from top of bedding). After 20 min exploration in the cage, mice were removed. The number of marbles that were at least 2/3 covered was counted.

Elevated plus maze (EPM): EPM test were conducted as previously described^46^^,^^47^. Briefly, a plus-cross-shaped plastic apparatus was used, which was elevated 58 cm from the floor and comprised two open and two closed arms (30 cm × 6 cm). Mice were placed in the center part of the maze facing one of the two open arms. Mice behavior was tracked for 10 min.

Open-field test (OFT): OFT were conducted as previously described^47^. Briefly, mice were gently placed in an open field, a white plastic box (46 cm × 46 cm × 40 cm). The center was located in 3/5 places of length and width. Mice were placed in the center of the arena tracked for 10 min.

### 2.6 UPLC–MS/MS

Tryptophan and Tryptophan metabolites were quantified by UPLC–MS/MS as done previously^47^. Briefly, tissue homogenate or bacterial supernatant was extracted with 70% methanol (Sinopharm, Shanghai, China), vortexed and centrifuged at 14,000×*g* for 15 min at 4 °C. Metabolites was separated and detected on an AB Sciex 4500 UPLC-MS/MS system (AB Sciex, CA, USA). Samples were injected (2 μL) and separated on a Waters BEH C18 column (Waters, Milford, USA) (100 mm × 2.1 mm × 1.7 mm). The mobile phase consisted of solution A (5 mmol/L ammonium formate, 0.1% formic acid) and solution B (0.1% formic acid in acetonitrile) at 0.3 mL/min at 40 °C. The gradient elution was programed as follows: 0–1 min, 95% A; 1–2 min, 95%–10% A; 2–4.5 min; 10% A; 4.5–4.6 min; 10%–95% A and 4.6–7 min, 95% A. All reagents were purchased from Sinopharm Chemical Reagent Co., Ltd. (Shanghai, China). Metabolites was detected using selected reaction monitoring of compound-specific mass transitions in positive electrospray ionization mode. Purity and mass of these metabolites was determined using ion-pair LC–MS (Supporting Information Table S2). Data acquisition and processing were performed with the analyst software MultiQuant 2.1.1.

### 2.7 Strains and media

All bacterial strains and plasmid used in this study are listed in Supporting Information Tables 3 and 4 EcN (non-pathogenic probiotic isolate, serotype O6: K5: H1) was kindly provided as a gift from Jun Zhu's lab (University of Pennsylvania). The detailed methods of DNA manipulation and genome editing for EcN are presented in the Supporting Information cell cultivation conditions were the same as previously reported^47^. Luria-Bertani (LB) medium with appropriate antibiotic selection–100 μg/mL streptomycin (BBI, Shanghai, China), 100 μg/mL ampicillin (BBI, Shanghai, China), 100 μg/mL kanamycin (BBI, Shanghai, China)–was used for cell cultivation. Cell growth was monitored by OD_600_ measurements. Modified M9 medium (M9Y) with 10 mM tryptophan (Sigma–Aldrich, MO, USA), 0.1% Casein Hydrolysate (BBI, Shanghai, China), 50 μg/mL FeCl_3_ (Sinopharm, Shanghai, China) and 0.2% ZYT [1.6% tryptone (Oxoid, Basingstoke, UK), 1% yeast extract (Oxoid, Basingstoke, UK), 0.5% NaCl (Sinopharm, Shanghai, China)] was used for production of 5-HTP and 5-HT in shake flasks. EcN_5-HT was incubated in LB at 37 °C overnight. Then, the medium was centrifuged at 8000×*g* for 10 min to obtain the supernatant. The production of 5-HT in fermentation supernatant was measured by UPLC–MS/MS.

### 2.8 EcN administration and quantification of bacterial colonization

MeCP2-TG mice were given antibiotic (ABX)-containing drinking water for 7 days before EcN administration. Then, the drinking water of the mice was replaced with normal water. Then, EcN WT, EcN_5-HTP, and EcN_5-HT (1 × 10^9^ CFU suspended in 100 μL of saline) were given through gavage every two days to the mice for 21 days, respectively, while mice from the normal group were treated with saline. This intermittent dosing protocol was adopted to minimize gavage-induced physiological stress^48^^,^^49^ while maintaining effective colonization, as demonstrated in previous probiotic and engineered bacteria studies^50^, ^51^, ^52^, ^53^, ^54^. After that, mice were used for behavioral testing and sacrificed for sample collection at the endpoint.

Fecal samples were collected to assess the intestinal colonization of EcN strains. Samples were homogenized in 1 × phosphate-buffered saline (PBS, BBI, Shanghai, China) at a 1:10 (*w*/*v*) ratio, and serial 10-fold dilutions were prepared in PBS. Aliquots (100 μL) of appropriate dilutions were plated onto selective LB agar plates in duplicate or triplicate and incubated at 37 °C for 16–24 h. Colonies were enumerated, and only plates containing 3–300 colony-forming units (CFU) were considered for quantification. Bacterial loads were expressed as CFU per gram of feces (CFU/g), with mean values and standard deviations calculated from replicate measurements for each time point.

### 2.9 Gastrointestinal (GI) symptom test

GI symptom tests were conducted as previously described^47^. Briefly, stool water content was calculated as Eq. (1):

(1) Stool water content (%) = [(Initial stool weight−Dry stool weight)/Initial stool weight] × 100

Stool frequency was measured as the number of stool pellets extruded from each mouse per hour. For the test of GI transit time, each mouse was gavaged with 0.2 mL of activated carbon solution (Rhawn, Shanghai, China) and sacrificed after 30 min. The GI transit time was determined by recording the length of the small intestine and the distance traveled by the activated carbon in the intestine.

### 2.10 16S rRNA gene sequencing and analyses

16S rRNA gene sequencing was performed as previously described^55^. Mice fecal samples were collected and frozen at −80 °C immediately. Total genomic DNA from approximately 200 mg of stool was extracted by a QIAamp DNA Stool Mini Kit (Qiagen, Hilden, Germany) according to the manufacturer's instructions. The V3–V4 region of the bacterial 16S ribosomal RNA (rRNA) genes was amplified by PCR with universal primers (338F, ACTCCTACGGGAGGCAGCAG; 806R, GGACTACHVGGGTWTCTAAT) and FastPfu Polymerase. Amplicons were then purified by gel extraction (AxyPrep DNA Gel Extraction Kit, Axygen, CA, USA) and quantified using QuantiFluor-ST (Promega, Wisconsin, USA). The purified amplicons were pooled in equimolar concentrations, and paired-end sequencing was performed using an Illumina MiSeq platform (Illumina, San Diego, USA).

Raw reads were first trimmed with Trimmomatic (v0.33)^56^ and primer sequences were recognized and removed from using Cutadapt software^57^. Subsequently, paired-end reads were assembled by Usearch (v10, http://www.drive5.com/usearch/). Microbial amplicon sequences were analyzed using QIIME2-DADA2 (v2020.6)^58^ to obtain non-chimeric reads and infer Amplicon Sequence Variants (ASV). Next, we used a naïve Bayesian classifier to assign taxonomic classes to ASVs based on Silva reference database (v138)^59^. Alpha and beta diversity analyses were conducted by QIIME2 and R packages.

### 2.11 Tracing experiment

For tracing experiment *in vivo*, eight weeks old MeCP2-TG male mice were divided into two groups and treated with oral gavage of either 5-HTP labeled with 3 deuterium atoms (5-HTP-d3, BePure, Shanghai, China) or 5-HT labeled with 4 deuterium atoms (5-HT-d4, BePure, Shanghai, China). At 0, 3, and 5 h after administration, three mice from each group were euthanized, and serum and brain tissue samples were collected and stored at −80 °C. UPLC–MS/MS metabolomics were performed to identify and quantify the presence of deuterium-labelled 5-HTP and 5-HT.

### 2.12 RNA-seq sequencing and analysis

Total RNA was extracted from mice tissue using Trizol reagent (Vazyme, Nanjing, China). RNA quantity was checked with Qubit 4.0 and RNA integrity was verified with Agilent Bioanalyzer 2100 (Agilent Technologies, CA, USA). Then, the mRNA is enriched by using the Hieff NGS® mRNA Isolation Master Kit (YEASEN, Shanghai, China) and the oligo(dT) magnetic beads. Sequencing was performed on DNBSEQ-T7 platform (MGI, Shenzhen, China) following manufacture's protocol. Reads were mapped to the hg19 reference genome using STAR 2.5, and gene expression was quantified with featureCounts^60^. Differential gene expression analysis was conducted through R using the DeSEQ2 package. Genes with a *P* value < 0.05 and a log_2_ (fold change) > 2 were assumed to be significantly differentially expressed. These differentially expressed genes were then subjected to enrichment analysis using KOBAS.13, with an FDR value of 0.05 employed as a cut-off threshold.

### 2.13 Golgi staining

Golgi staining was performed using Golgi-Cox OptimStainTM kit in accordance with the manufacturer's instruction. In brief, sliced brain samples were fixed with formaldehyde, rehydrated, and embedded in paraffin. Sections of brain were stained with staining solution for 10 min at room temperature. The images were taken with microscopy (Olympus BX-50, Japan). Number of branches and branch length of neurons in the hippocampus were analyzed using ImageJ software by an observer blinded to the experiments.

### 2.14 Histological analysis

For immunofluorescence staining, frozen slices of the dissected brain tissues from different groups were blocked with 5% BSA (Beyotime, Shanghai, China) in PBS for 60 min. Heat mediated antigen retrieval was performed in 0.01 mol/L citrate-buffer (pH 6.0). The slices were then incubated with a 1:500 dilution of anti-5-HTP antibody, anti-5-HT antibody or anti-Iba1 antibody (Abcam, Cambridge, UK). Then, the slices were rocked on an orbital shaker (Mini Roller, NEST, Wuxi, China) at 4 °C in the dark overnight. Afterwards the slices were treated with HRP-conjugated secondary antibody (Abcam, Cambridge, UK), in PBS at room temperature in the dark for 60 min. Cell nuclei were stained with DAPI (Sigma−Aldrich, MO, USA). Stained cells were then visualized by fluorescence microscopy (Nikon Eclipse CI, Japan).

### 2.15 Western blot

Hippocampal tissue was lysed in 10 times the volume of tissue lysate containing protease and phosphatase inhibitors. Two 4 mm steel beads were added, and samples were homogenized for 2 min using a tissue homogenizer. After 30 min incubation on ice, lysates were centrifuged at 12,000 rpm for 10 min at 4 °C. The supernatant was collected, and total protein was quantified using a BCA protein assay kit (Beyotime, Shanghai, China). Then, protein extracts were mixed with the reducing sample buffer and heated in boiling water for 15 min. Next, samples were resolved by SDS-PAGE and transferred to PVDF membrane. Subsequently, the membrane was blocked for 1 h at room temperature with 5% milk solution (or 5% BSA for phosphorylated proteins detection) in TBS-Tween (Tris-buffered saline [10 mmol/L Tris, 5.8 g/L NaCl, pH 7.5] containing 0.5% Tween-20) and incubating with primary antibody including GAPDH antibody (AF7021, Affinity, Jiangsu, China), ERK1/2 Antibody (AF0155, Affinity, Jiangsu, China), Phospho-ERK1/2 (Thr202/Tyr204) Antibody (AF1015, Affinity, Jiangsu, China), TrkB antibody (AF6461, Affinity, Jiangsu, China), Phospho-Trk B (Tyr515) antibody (abs131087, Absin, Shanghai, China), EGFR antibody (AF6452, Affinity, Jiangsu, China), and Phospho-EGFR (Ser1070) antibody (AF3044, Affinity, Jiangsu, China) at 4 °C overnight. Then, the membranes were incubated with 1:5000 diluted Anti-Rabbit IgG HRP antibody (ab6721, Abcam, Cambridge, UK) for 1 h. Chemiluminescent signals in protein bands were detected with an IVIS imaging system.

### 2.16 Gene expression

qPCR was performed to examine gene expression as previously described^55^. Briefly, RNA from harvested brain tissues was extracted with TRIzol reagent (Invitrogen, CA, USA). HiScript II 1st Strand cDNA Synthesis Kit (Vazyme, Nanjing, China) was used to generate cDNA, and CFX Connect Real-Time PCR Detection System (Bio-Rad) was used to measure mRNA relative expression. PCR was carried out with 10 μL of SYBR Green Master Mix (Yeasen, Shanghai, China), 2 μL of complementary DNA (cDNA), 0.4 μL of forward primer, 0.4 μL of reverse primer, and 7.2 μL of nuclease-free water. The samples were subjected to 40 cycles of amplification. Preincubation was for 30 s at 95 °C, followed by denaturation at 95 °C for 10 s, annealing at 58 °C for 20 s, and extension at 72 °C for 30 s. The primers used in the present study are listed in Supporting Information Table S5.

### 2.17 Hippocampal electroencephalographic (EEG) signal processing and spectral analysis

Mice were anesthetized with 1.5%–2.0% isoflurane (Sigma−Aldrich, MO, USA) and placed in a stereotaxic frame under aseptic conditions. Stainless-steel bipolar electrodes (125 μm diameter; Plastics One) were implanted into the dorsal hippocampus (coordinates from bregma: AP −2.0 mm, ML ±1.5 mm, DV −1.4 mm). Reference and ground electrodes were placed over the frontal cortex and cerebellum, respectively. All electrodes were secured to a micro-connector pedestal (EIB-16) with dental acrylic and covered with a custom Faraday cage to minimize electrical noise. EEG signals were amplified 1000 × and digitized continuously at 2 kHz with a 0.1–500 Hz band-pass filter using a Digital Lynx 16-channel acquisition system (Neuralynx, Montana, USA). Simultaneous video was recorded at 30 frames per second and synchronized to the electrophysiological data *via* TTL pulses.

### 2.18 Statistical analysis

Statistical analyses were performed using Origin software (OriginLab). Data normality was verified by Shapiro-Wilk tests, with homogeneity of variance confirmed by Levene's test. Parametric tests (unpaired two-tailed *t*-tests for two groups; one-way ANOVA for multiple groups) were applied when assumptions were met; otherwise, Mann-Whitney U tests were used. All data are presented as mean ± SD unless otherwise noted. Significance thresholds were maintained at ∗*P* < 0.05 and ∗∗*P* < 0.01.

## 3 Results

### 3.1 Low level of 5-HTP in ASD children and mouse models

**Figure 1:**
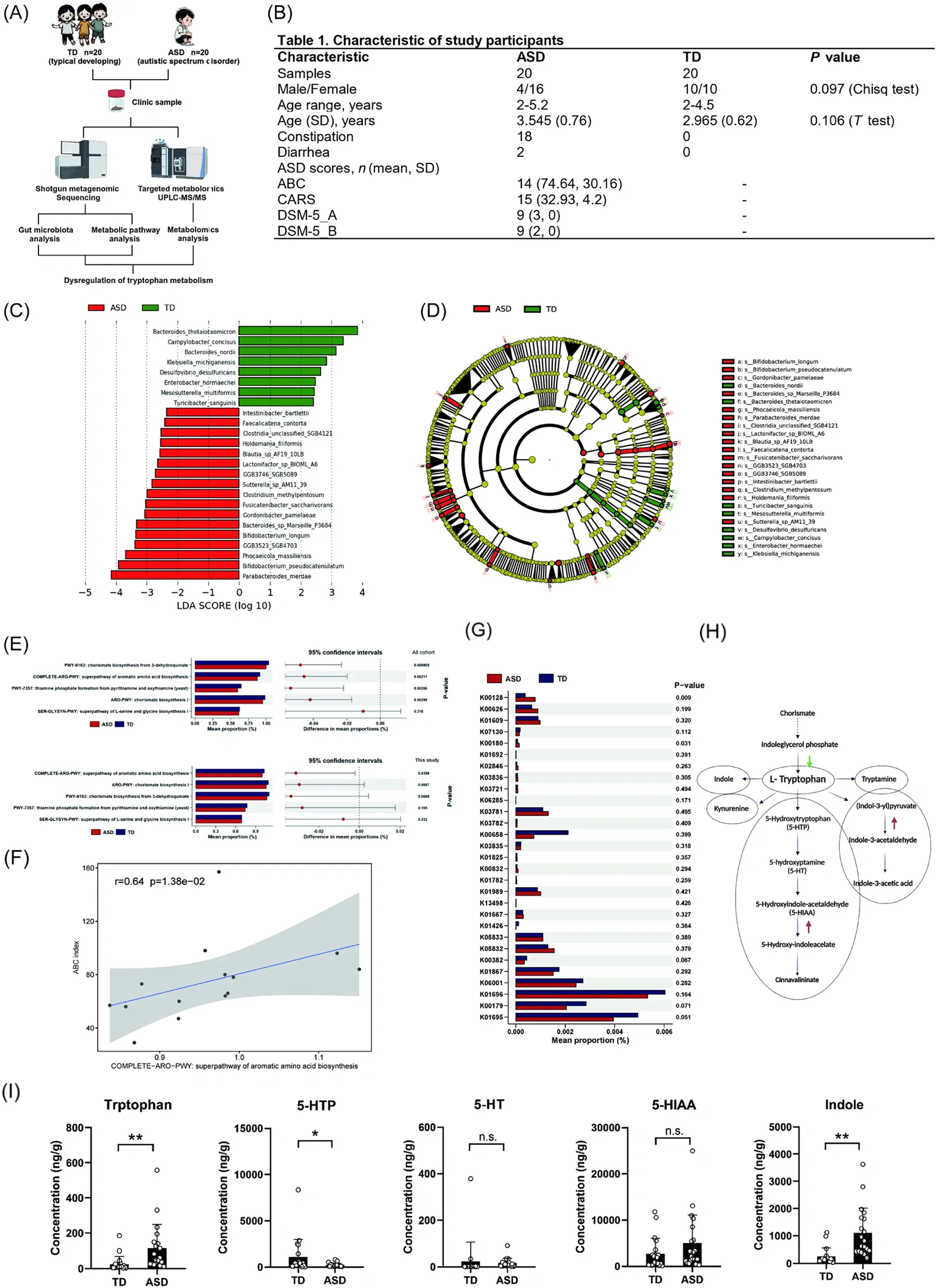
Multi-omic analysis corroborates tryptophan biosynthesis and metabolism is reprogramed in ASD children. (A) Experimental design. (B) Characteristic of study participants. (C) LDA effect size (LEfSe) cladogram of taxonomic data of fecal samples from TD (Green) and ASD (red) group (*P* < 0.05; LDA score 2.0). (D) Cladogram of LEfSe linear discriminant analysis of microbiome from TD and ASD patients. Green and red circles represent the differences of the most abundant microbiome class. The diameter of each circle is proportional to the relative abundance of the taxon. (E) Aromatic amino acid biosynthesis pathway enriched in the microbiota of ASD children by Gene Set Enrichment Analysis (GSEA) in four public datasets and this study. (F) Spearman correlation analysis of aromatic amino acid biosynthesis pathway and ABC score. (G) Genes involved in tryptophan biosynthesis and metabolism enriched in the microbiota of ASD children. (H) Schematic diagram summarizing the changes in tryptophan biosynthesis and metabolism in ASD children. (I) Contents of tryptophan metabolites in fecal samples of TD and ASD children. Mean values ± SDs are presented (*n* = 20), *P* values were calculated using unpaired *t*-test, ∗*P* < 0.05 and ∗∗*P* < 0.01. n.s: not significant.

We collected and analyzed stool samples from 20 unmedicated ASD children and 20 typically developing children (Fig. 1A and B, Supporting Information Table S1). There were no significant differences in key demographic characteristics, including gender and age between the two groups of children. These children were tested with the Autism Behavior Checklist (ABC), the Childhood Autism Rating Scale (CARS), and the Diagnostic and Statistical Manual of Mental Disorders (DSM-5) to assess the existence and severity of autism (Fig. 1B). To characterize the microbiome profile in ASD, stool samples from ASD children were subjected to metagenomic sequencing. Compared with healthy children, ASD children showed significantly elevated Shannon diversity in the fecal microbiome (*P* = 0.045, Supporting Information Fig. S1A). Although the NMDS (Nonmetric multidimensional scaling) analysis indicated that the fecal microbiome from ASD children and healthy children clustered together (Fig. S1B), the LEfSe analysis detected 25 differentially abundant species between the two groups (LDA >2.0, Fig. 1C and D). FMT experiments have proved that the disordered gut microbiome from ASD children induced the development of social novelty deficiency (Fig. S1C–D′), repetitive behaviors (Fig. S1E–E′), and anxiety symptoms (Fig. S1F–G′). These results demonstrate that the composition of gut microbiota is associated with the symptoms of autism, consistent with previous research findings^23^^,^^37^.

We further performed functional pathway prediction based on the metagenomic data to explore the interaction between the host and its gut microbiota. Across four public datasets and our study, the aromatic amino acid biosynthesis pathway was significantly enriched in the gut microbiota of TD children relative to ASD children (Fig. 1E), and the pathway's abundance was significantly correlated with the severity of ASD (Fig. 1F). Among the aromatic amino acids, tryptophan (Trp) metabolites are the most studied categories of metabolites involved in host-microbiota interactions^61^. To investigate the changes in tryptophan metabolites, we compared all the high-throughput sequencing reads of the present study involved in the tryptophan biosynthesis and metabolic pathway. In the microbiota from ASD children, the gene encoding tryptophan synthase (TRPA, K01695) was significantly down-regulated, while the gene encoding the enzyme that metabolizes 5-hydroxyindole-acetaldehyde to 5-hydroxy-indoleacetic acid (ALDH, K00128) and the enzyme that metabolizes (indol-3-yl) pyruvate to Indole-3-acetaldehyde (IORB, K00180) were significantly up-regulated in the ASD group (Fig. 1G and H).

Together with our data, these results suggested that the intermediate metabolites in the 5-HT pathway may play important roles in ASD physiological and pathological processes. To further quantify these tryptophan metabolites in the gut, we performed targeted metabolomics. Using UPLC–MS/MS data and chemical standards, we found that the levels of three tryptophan-related metabolites were significantly altered in the fecal samples from ASD children: tryptophan, 5-HTP, and indole (Fig. 1I). Marked elevation in tryptophan and its metabolite indole were shown, while 5-HTP production was significantly reduced in fecal samples from ASD children. Metabolites downstream of 5-HTP (5-HT and 5-HIAA) did not differ significantly by groups (Fig. 1I). Metabolic flux analysis revealed a shift toward indole production in ASD children.

In MeCP2-TG (human methyl CpG binding protein 2 transgenic) autism model mice^40^^,^^41^^,^^62^ we observed gastrointestinal motility disturbances (Supporting Information Fig. S2A) and an altered gut community distinct from normal mice (Fig. S2B and S2C). Long-term exposure to bacteria from normal mice could reverse the gastrointestinal and behavioral impairments of MeCP2-TG mice (Fig. S2D–S2I). We also observed that tryptophan metabolism shifted from the 5-HTP pathway to the indole and kynurenine pathways in fecal and serum samples from MeCP2-TG mice (Fig. S2J).

### 3.2 Microbiota-derived 5-HTP modulates behavioral abnormalities in different ASD mouse models

**Figure 2:**
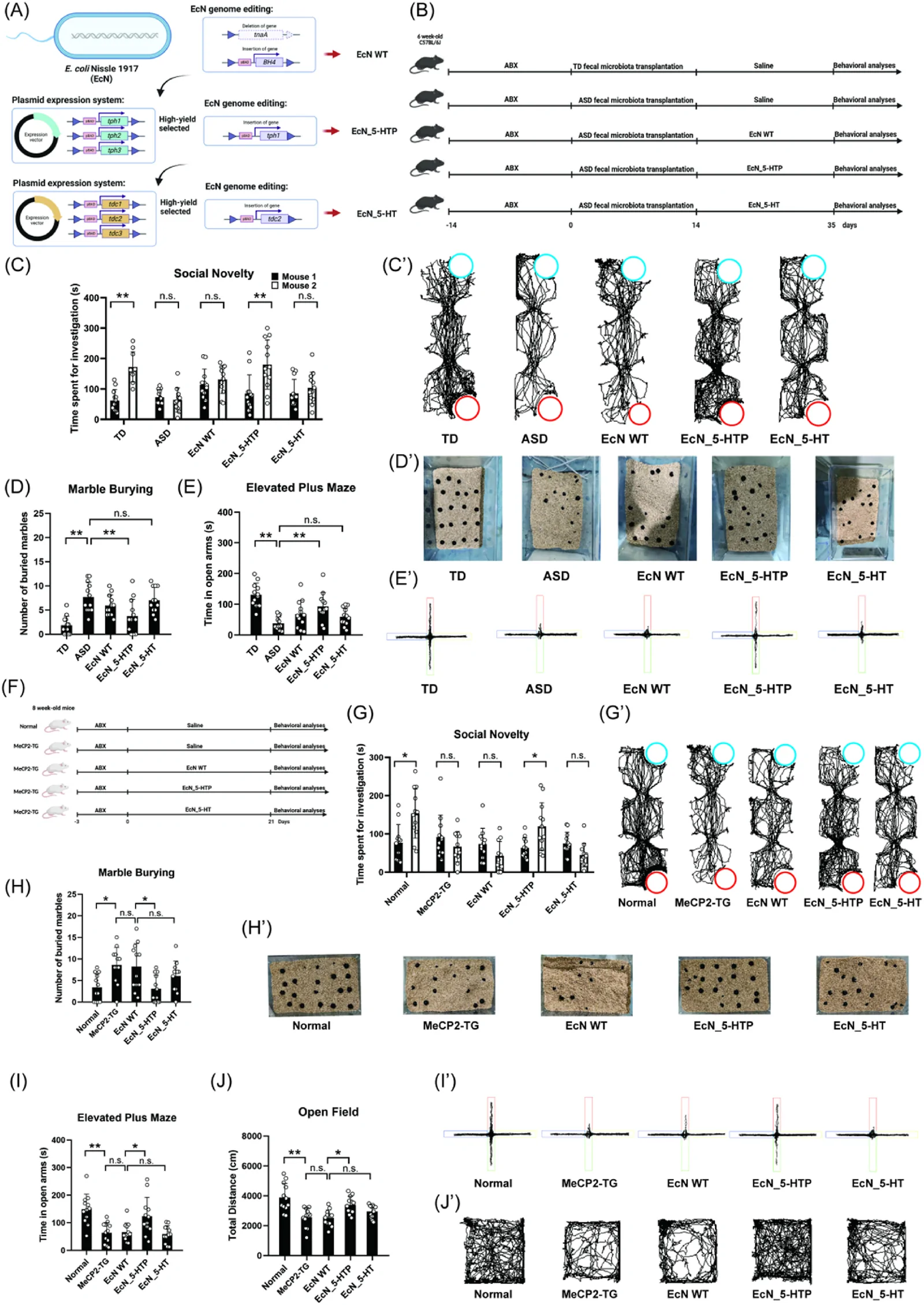
Microbiota-derived 5-HTP modulate behavioral abnormalities in mice models. (A) Engineer *Escherichia coli* Nissle 1917 (EcN) to synthesis of 5-HTP and 5-HT. (B) Experimental setup of FMT mice model. (C–C′) 3-chamber sociability index. (D–D′) Repetitive behavior by marble burying test. (E–E′) Entries and time spent in open arms by elevated plus maze. (F) Experimental setup of MeCP2-TG mice model. (G–G′) 3-chamber sociability index. (H–H′) Repetitive behavior by marble burying test. (I–I′) Entries and time spent in open arms by elevated plus maze. (J–J′) Distance traveled by open field testing. Mean values ± SDs are presented (*n* = 12), *P* values were calculated using unpaired *t*-test, ∗*P* < 0.05 and ∗∗*P* < 0.01. n.s: not significant.

*Escherichia coli* is generally a normal commensal of humans, being one of the first colonizers of our guts^63^. The human endogenous gut bacteria *E*. *coli* Nissle 1917 (EcN) was selected as a chassis strain due to its established clinical safety profile^64^^,^^65^ and experimental tractability. To investigate the function of microbiota-derived 5-HTP and 5-HT, we used EcN as the microbial delivery platform for 5-HTP and 5-HT production to mimic the physiological environment *in vivo* (Fig. 2A). 5-HTP is natively produced from l-tryptophan by tryptophan hydroxylase (TPH) in humans and animals^61^. To improve the l-tryptophan availability, we knocked out the *tanA* gene in EcN *via* a CRISPR-Cas9 system^66^ (Supporting Information Fig. S3A). The resulting EcN Δ*tanA* strain lacks the enzyme for indole synthesis from l-tryptophan (Fig. S3B). Then, the 5-HTP biosynthetic pathway was introduced into EcN (Fig. S3C and S3D). 5-HTP production in EcN_5-HTP culture supernatants reached 1786.61 ng/mL after 24-h of incubation. Then, TDC from *Oryza sativa*, which resulted in the highest yield of 5-HT in EcN, was selected to be integrated into the EcN_5-HTP genome^47^. A yield of 80.6 mg/L 5-HT was achieved after 24-h incubation of the recombinant strain (EcN_5-HT) (Fig. S3E).

Next, we administered the EcN WT, EcN_5-HTP, and EcN_5-HT separately to the mice transplanted with the microbiota from the ASD children (*n* = 12/group) (Fig. 2B). Monitoring confirmed that the administered strains could stably persist in the murine gut throughout the gavage intervention period while maintaining consistent 5-HTP/5-HT production *in vivo* (Fig. S3F–S3H). As shown in Fig. 2C–C′, EcN_5-HTP ameliorated impaired social novelty of ASD-FMT mice during the three-chambered social test. Compared with ASD-FMT mice treated with saline or EcN WT, mice treated with EcN_5-HTP spent more time with the novel mouse, suggesting EcN derived 5-HTP may play a role in social preference (Fig. 2C–C′). Further analysis showed that mice treated with EcN_5-HTP also exhibited less marble-burying behavior (Fig. 2D–D′). Besides, mice in EcN_5-HTP group spent notably more time in the open arms in the EPMT (Fig. 2E–E′). Together, these results indicate that EcN_5-HTP but not EcN_5-HT ameliorated autistic related behavioral abnormalities induced by dysbiotic microbiota, suggesting that microbe derived 5-HTP can improve autistic symptoms and exert anxiolytic effects in host gastrointestinal tract (Fig. 2C–E′).

**Figure 3:**
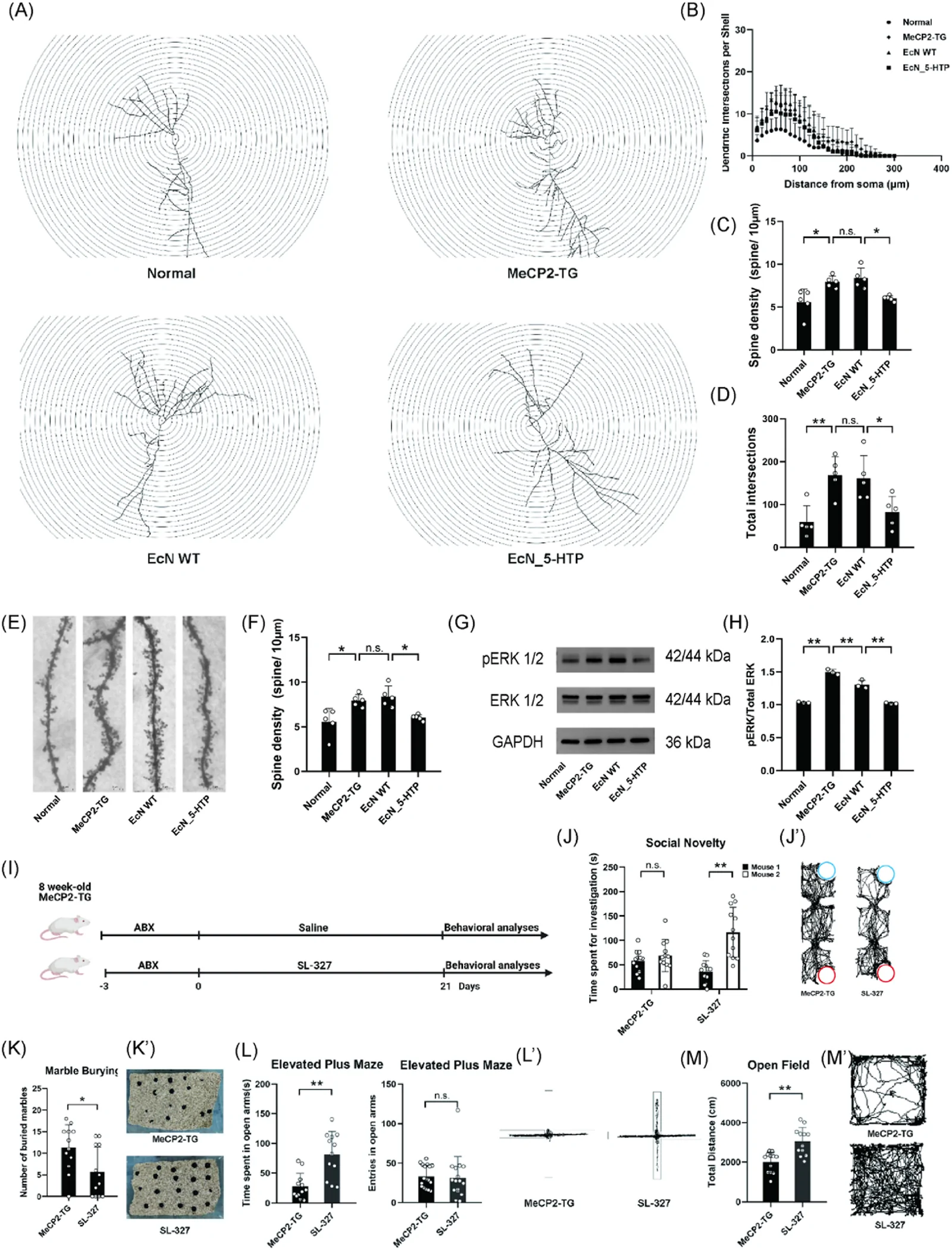
Microbiota-derived 5-HTP ameliorates dendritic spines overproduction through ERK Pathway. (A) Representative images of hippocampus neurons superimposed over concentric used for Sholl analyses. (B–D) Quantification of dendritic branch complexity by Sholl analysis. (E) Representative images of dendritic spines. (F) Measurements of spine density over 10 μm dendrites. (G–H) Western blots of activated ERK and total ERK in hippocampus. (I) Experimental setup. (J–J′) 3-chamber sociability index. (K–K′) Repetitive behavior by marble burying test. (L–L′) Entries and time spent in open arms by elevated plus maze. (M–M′) Distance traveled by open field testing. Mean values ± SDs are presented (*n* = 12), *P* values were calculated using unpaired *t*-test, ∗*P* < 0.05 and ∗∗*P* < 0.01. n.s: not significant.

To test 5-HTP effects on host, antibiotic-treated MeCP2-TG mice received oral administration of EcN WT, EcN_5-HTP, and EcN_5-HT (1 × 10^9^ CFU every two days) for two weeks (Fig. 2F). As shown in Fig. 2G–G′, MeCP2-TG mice spent significantly less time interacting with a novel mouse over a familiar mouse in the social novelty test compared to normal mice, indicating that they had a deficit in social novelty behavior. On the other hand, the social approach behavior of MeCP2-TG mice indicated by the time interacting with a stranger mouse over a toy was basically normal (Fig. S3F), which is consistent with previous results^41^. MeCP2-TG mice also exhibit impaired repetitive behaviors (Fig. 2H–H′) in the marble-burying test and impaired exploration behavior in the elevated plus maze (Fig. 2I–I′) and open field (Fig. 2J–J′). The administration of EcN_5-HTP increased the percentage of time that the mice spent interacting with a stranger mouse (Fig. 2G–G′). EcN_5-HTP also reduced marble burying in MeCP2-TG mice (Fig. 2H–H′). On the EPM, animals treated with EcN_5-HTP had a larger number of entries to the open arms and more time spent in the open arms than EcN WT-fed animals (Fig. 2I–I′). Treatment with EcN_5-HTP also modulated locomotor activity in the open field and restored the mobility of transgenic mice to control levels (Fig. 2J–J′). Consistent with the results in the human FMT mouse model, there was no autism-related behaviours treatment effect observed in the EcN_5-HT group (Fig. 2G–J′). The 16S rRNA sequencing revealed no significant *β*-diversity differences or outlier clustering between EcN WT and EcN_5-HTP groups (Fig. S3I), indicating that the engineered strain's therapeutic effects primarily result from its synthetic function rather than microbiota ecological alterations. Considering that a variety of factors contribute to the development of ASD, we try to explore the effect of a high-yielding 5-HTP enterobacterial intervention in another mouse model of autism. The BTBR inbred mice is a commonly used animal model for spontaneous autism. We repeated all those experiments in BTBR mouse model. Even though EcN_5-HTP did not improve BTBR social behaviors, it significantly ameliorated repetitive stereotyped behavior and anxiety-like behavior (Supporting Information Fig. S4). Collectively, these results indicate that microbiota-derived 5-HTP plays a role in regulating the status of ASD-related behaviours.

Moreover, we directly administered 5-HTP orally to MeCP2-TG mice and found that oral administration of 5-HTP enhanced social behaviours in the mice (Supporting Information Fig. S5A–B′). However, 5-HTP did not significantly alleviate repetitive stereotyped behaviors in the marble burying test (Fig. S5C–C′), but significantly improved anxiety-like behaviours in the elevated plus maze test and the open field test (Fig. S5D–E′).

### 3.3 Microbiota-derived 5-HTP ameliorates dendritic spine overproduction *via* ERK pathway

Aberrant dendritic development has been identified as a distinctive characteristic of neurodevelopmental anomalies in individuals with ASD^67^. Perturbations in both the density and morphology of dendritic spines within specific brain regions are believed to underlie alterations in synaptic function and impairments in neuronal information transmission in ASD pathogenesis^68^. Consistent with preceding investigations, the MeCP2-TG mice exhibited augmented complexity of dendritic arborization and excessive dendritic spine formation within the hippocampal region compared to their control counterparts (Fig. 3A–D). Remarkably, intervention with EcN_5-HTP resulted in the restoration of aberrant dendritic morphology and a concomitant reduction in dendritic spine density to normal levels (Fig. 3E and F). These findings suggest a putative influence of gut microbiota-derived 5-HTP on dendritic development. Since the MAPK/ERK signaling pathway is closely associated with neuron dendritic development in ASD^69^, ^70^, ^71^, we focused on the MAPK/ERK signaling pathway and assessed its activity. As anticipated, the model mice exhibited excessive activation of the ERK pathway, while treatment with EcN_5-HTP resulted in appropriate suppression of ERK over-phosphorylation (Fig. 3G and H). RNA sequencing analysis also predicted that the ameliorative effect of 5-HTP on autism-related behaviors was associated with the MAPK/ERK signaling pathway (Supporting Information Fig. S6).

In order to validate the impact of the ERK pathway on behaviors related to autism, we administered the ERK inhibitor SL-327 to the mice. After three weeks administration of SL-327 (20 mg/kg) *via* intraperitoneal injection, behavioral tests were conducted (Fig. 3I). As shown in Fig. 3, the application of the ERK inhibitor to MeCP2-TG mice resulted in a significant improvement in social novelty (Fig. 3J–J′), as well as a reduction in repetitive behaviors (Fig. 3K–K′) and anxiety-like behaviors (Fig. 3L–M′).

### 3.4 Inhibition of RTK ligand expression and RTK receptor activity by microbiota-derived 5-HTP

**Figure 4:**
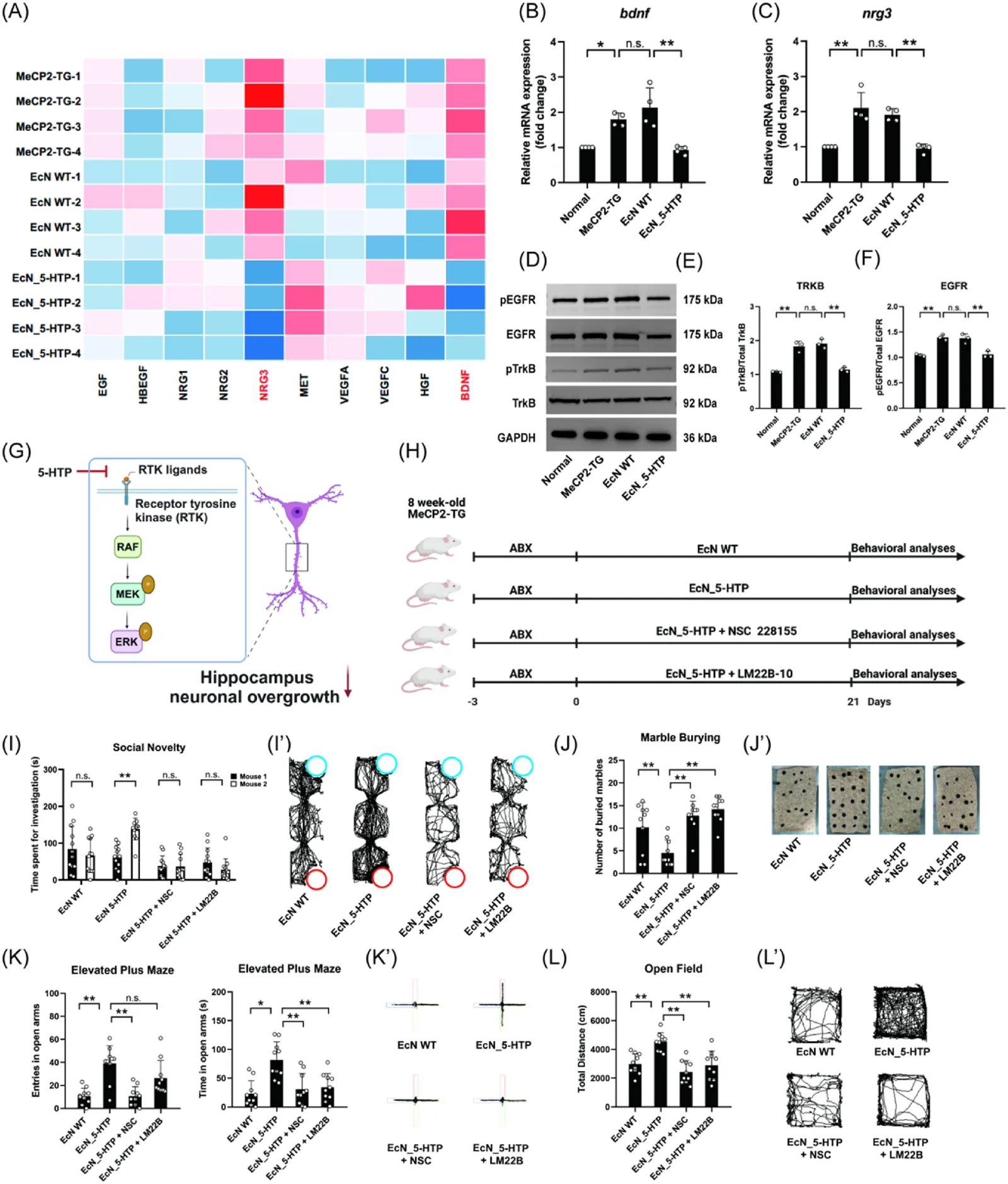
Microbiota-derived 5-HTP suppresses the expression of RTP ligands and inhibits the activity of RTK receptors. (A) Heat map showing the expression level of RTK ligand genes. (B, C) qPCR analysis of *nrg3* and *bdnf* mRNA expression. (D–F) Western blots of activated EGFR and TrkB. (G) The function of 5-HTP on RTK/ERK pathway. (H) Experimental setup. (I–I′) 3-chamber sociability index. (J–J′) Repetitive behavior by marble burying test. (K–K′) Entries and time spent in open arms by elevated plus maze. (L–L′) Distance traveled by open field testing. Mean values ± SDs are presented (*n* = 10), *P* values were calculated using unpaired *t*-test, ∗*P* < 0.05 and ∗∗*P* < 0.01. n.s: not significant.

Given the pivotal role played by RTK receptors in the upstream regulation of the ERK pathway, we postulated that 5-HTP might influence the ERK pathway through the RTK receptors. To explore this further, we investigated the expression of ligand genes of the RTK receptor family and observed a downregulation of BDNF and NGR3 upon 5-HTP treatment (Fig. 4A–C). Subsequently, we evaluated the activation status of the TrkB receptor and the EGFR receptor. Remarkably, our results revealed that intervention with EcN_5-HTP led to a robust attenuation of excessive phosphorylation of both TrkB receptors and EGFR receptors within the hippocampal region (Fig. 4D–F). To ascertain whether gut microbiota-derived 5-HTP modulates host behavior *via* the RTK receptor-mediated signaling cascade (Fig. 4G), we co-administered EcN_5-HTP to MeCP2-TG mice while concurrently injecting them with a TrkB receptor activator LM22B-10 and an EGFR receptor activator NSC 228155. To maximize compound brain levels, mice received LM22B-10 through combined administration of 22 mg/kg intraperitoneally and 10 mg/kg intranasally^72^. NSC 228155 was delivered intracerebrally to the hippocampal dentate gyrus following established protocols^73^. Subsequently, behavioral tests were conducted at the experimental endpoint (Fig. 4H). Intriguingly, the findings demonstrated that activation of either receptor alone effectively abolished the ameliorative effects of gut-derived 5-HTP on autism-like behaviors (Fig. 4I–J′) and anxiety-like behaviors (Fig. 4K–L′). These results indicate that the ameliorative effects of gut microbiota-derived 5-HTP on autism-related behaviors are mediated by downregulation of RTK ligand expression as well as suppression of the downstream RTK receptor/MAPK/ERK signaling cascade.

### 3.5 5-HTP crosses the blood–brain barrier and affects host 5-HTP pools

**Figure 5:**
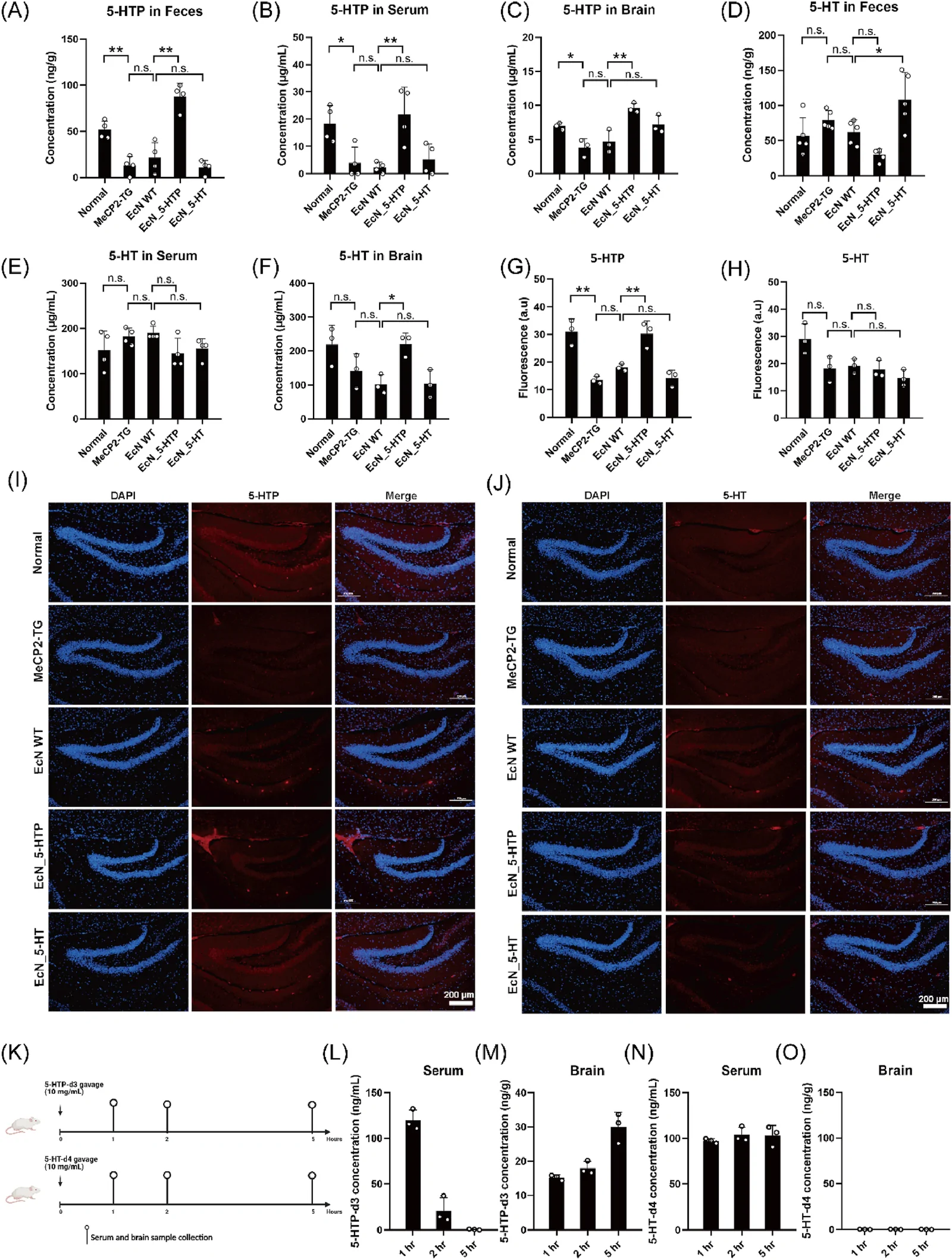
Microbiota-derived 5-HTP could cross the blood-brain barrier and affect host 5-HTP pool in the brain. (A–F) Contents of 5-HTP and 5-HT in fecal, serum and whole-brain homogenate samples. (G–H) Quantitation of 5-HTP and 5-HT fluorescence intensity in the hippocampus. (I) Fluorescent microscope pictures of the hippocampus showing 5-HTP antibody staining (red) and cell nuclei (blue). (J) Fluorescent microscope pictures of the hippocampus showing 5-HT antibody staining (red) and cell nuclei (blue). (K) Deuterium labeling experiments. (L–M) The deuterium labeled 5-HTP in serum and brain sample after gavage. (N–O) The deuterium labeled 5-HT in serum and brain sample after gavage. Mean values ± SDs are presented (*n* = 3), *P* values were calculated using unpaired *t*-test, ∗*P* < 0.05 and ∗∗*P* < 0.01. n.s: not significant.

Several studies have suggested that oral 5-HTP may penetrate the blood–brain barrier (BBB)^74^^,^^75^. Therefore, we conjecture that 5-HTP from gut may have reached the central nervous system *via* the somatic circulation, which in turn affects neuronal development. To test that, blood and fecal samples from mice in different groups were collected and assayed for metabolites analysis. The results showed that EcN_5-HTP treatment leads to an increase of 5-HTP in feces (Fig. 5A), serum (Fig. 5B), and brain (Fig. 5C). After EcN_5-HT administration, the level of 5-HT in serum and brain showed no significant difference compared with EcN WT group although the level of 5-HT in feces increased (Fig. 5D–F). Further, we examined the levels of 5-HTP and 5-HT in the hippocampal region using immunofluorescence. The quantitative results showed that 5-HTP levels in the hippocampal region of mice gavaged with EcN_5-HTP were significantly higher compared to mice gavaged with EcN WT (Fig. 5G–I), while gavage of EcN_5-HT had little effect on the 5-HT levels in the hippocampal region of mice (Fig. 5H–J). These results are consistent with the results of mass spectrometry assay and also predict that there may be a difference in the ability of 5-HTP and 5-HT of enterobacterial origin to cross the host BBB.

To validate this, tracing experiments were conducted using the deuterium labelled 5-HTP and 5-HT (Supporting Information Fig. S7A). As shown in Fig. 5K, mice were dosed with 5-HTP-d3 or 5-HT-d4 by oral gavage and sacrificed after 1, 2, and 5 h. Then, serum and brain samples were collected, and the deuterium labelled 5-HTP and 5-HT were determined by UPLC–MS/MS. 5-HTP-d3 was already detectable in serum and brain tissue 1 h after gavage. Following gavage, the concentration of 5‑HTP‑d3 in serum decreased progressively over time (Fig. 5L), suggesting its clearance from the circulation. Meanwhile, the level of labelled 5‑HTP in the brain increased with time, reaching a mean concentration of 30 ng/mL at 5 h post‑gavage (Fig. 5M), suggesting that the 5-HTP flow through the systemic circulation path to the brain. In contrast, 5‑HT‑d4 administration showed no change in serum levels after gavage (Fig. 5N) and was not detectable in brain tissue at any examined time point (Fig. 5O). After gavage of the deuterium markers in wild-type normal mice, there was a consistent trend for the markers to reach serum and brain tissues at different time points (Fig. S7B–E), indicating that the ability of 5-HTP to cross the BBB was not related to the disease state of MeCP2-TG mice. The effect of microbiota-derived 5-HTP on host behaviors might be associated with its capacity to penetrate the BBB.

## 4 Discussion

In recent years, the prevalence of ASD has been increasing worldwide. According to data released by the Autism and Developmental Disabilities Monitoring Network in the United States, the incidence of autism has risen from 1 in 150 to 1 in 36 over the past 20 years^76^. ASD is a clinically heterogeneous class of neurodevelopmental disorders whose pathogenesis is currently unclear. The risk factors for ASD include genetics, environment, prenatal exposure, immune, nutrition, etc.^77^^,^^78^. The discovery that the gut microbiota may operate as a fundamental regulator of the brain and behaviors of the host has been one of the most intriguing findings in recent years^79^. Many studies have revealed the therapeutic potential of targeting the microbiome in treating autism^34^^,^^37^^,^^38^^,^^80^, ^81^, ^82^, ^83^, ^84^. In the present study, the tryptophan metabolite, 5-HTP, has been identified as a potential link between gut microbiota and ASD. Our study demonstrates that gut microbiota-derived 5-HTP crosses the blood–brain barrier and influences dendritic spine development, suggesting a direct interaction between gut metabolites and the nervous system that alleviates ASD symptoms in various mouse models.

As an established probiotic strain, EcN has been shown to exert beneficial effects through multiple mechanisms^85^, ^86^, ^87^. Our LC–MS/MS analyses confirmed that only EcN_5-HTP (not EcN WT) significantly increased 5-HTP/5-HT levels in both gut and brain tissues (Fig. 5A–H). This demonstrates that the additional behavioral improvement seen with EcN_5-HTP (*vs*. EcN WT) is indeed attributable to microbial 5-HTP production. These data confirm that while EcN itself may have mild baseline effects, the superior behavioral rescue by EcN_5-HTP is specifically mediated through 5-HTP-dependent mechanisms.

Metagenomic data from the clinical samples we collected showed that the structure and composition of gut microbiota in children with autism differ from those of typically developing children. The LEfSe analysis detected 25 differentially abundant species between the gut microbiota from ASD and TD (LDA >2.0, Fig. 1C and D). Strains such as *Parabacterioides merdaes*, *Bifidobacterium pseudocatenulatum*, and *Phocaeicola massiliensis* were significantly enriched in the autism group, while strains such as *Bacteroides thetaiotaomicrons*, *Campylobacter conicus*, and *Bacteroides nordiis* were significantly enriched in the control group. Notably, *Bacteroides thetaiotaomicron*–whose reduced abundance has been documented in both BTBR and Shank3 KO mouse models of ASD^88^^,^^89^–showed a strong positive association with tryptophan synthase (TRPA, K01695) expression (Supporting Information Fig. S8A), suggesting its potential role in promoting 5-HTP biosynthesis. Concurrently, we observed significant upregulation of aldehyde dehydrogenase (ALDH, K00128), the key enzyme responsible for 5-HTP conversion to 5-HIAA, which demonstrated pronounced negative correlations with both *Bifidobacterium longum* and *Eggerthella lenta* (Fig. S8A). Together, these microbial metabolic perturbations appear to shunt tryptophan flux toward alternative pathways, particularly indole production, potentially accounting for the characteristic 5-HTP deficiency and indole accumulation hallmarking ASD.

Recent advances in understanding ASD have highlighted the multifactorial etiology involving genetic, environmental, and prenatal factors. To validate the broad applicability of our findings in ASD treatment, we employed multiple mouse models of autism: the fecal microbiota transplantation model, transgenic ASD model (MeCP2-TG mice), and spontaneous ASD model (BTBR mice). Consistent results were obtained between the fecal microbiota transplantation and transgenic models, demonstrating that gut microbe-derived 5-HTP modulates ASD-related behavioral abnormalities (Fig. 2). In the BTBR mice model, while EcN_5-HTP did not improve social behaviors, it significantly ameliorated repetitive stereotyped behavior and anxiety-like behavior (Fig. S4). These findings suggest that 5-HTP may have broad clinical implications for various behavioral disorders. The reason why 5-HTP has different effects in different mouse models may be due to the different causes of ASD related symptoms in these animal models. The appropriate dosage of MeCP2 is crucial, as both MeCP2 underexpression and overexpression can lead to an autistic phenotype in some individuals^90^. The increase in the formation and stabilization of dendritic spines in the cerebral cortex of the MeCP2-TG mouse model contributes to the phenotypes in autism-like behaviors^91^. Our data indicated that the ameliorative effects of gut microbiota-derived 5-HTP on autism-related behaviors may be mediated by the suppression of the RTK/MAPK/ERK signaling cascade and reversal of the dendritic spines' overproduction. Our findings suggest that 5-HTP supplementation inhibits RTK ligand expression in the ASD model (Fig. 4A–F). While the precise mechanisms of 5-HTP's regulation of neurotrophic factors remain incompletely characterized, existing evidence suggests serotonergic signaling may play a mediating role. The observed prevention of stress-induced BDNF downregulation by 5-HT_2A_ receptor antagonists^92^ implies that 5-HTP might similarly modulate neurotrophic expression through GABAergic interneuron activity and subsequent transcriptional regulation. These potential mechanisms, while requiring further experimental validation, suggest an important intersection between serotonin metabolism and neurodevelopmental signaling pathways that warrants deeper investigation in ASD models. Although the mechanism underly is unclear, BTBR mice have an increased susceptibility to autism. Research has found that neurogenesis in the hippocampus of adult BTBR mice is significantly reduced, with a decrease in lateral ventricle volume and abnormal changes in neuroimmune responses within the brain^93^^,^^94^. However, we have not delved into the specific mechanism by which 5-HTP produced by gut microbiota affects the behavior of BTBR mice. Considering the diverse etiological factors associated with ASD, personalized interventions are needed for the treatment of different types of ASD patients.

Our results demonstrated that microbe-derived 5-HTP successfully improves autism-like behaviors and anxiety-like behaviors in murine models (Fig. 2). On the other hand, following oral gavage of 5-HTP reagent in MeCP2-TG mice, improvements were observed in social and anxiety-like behaviors, but repetitive stereotyped behaviors were not alleviated (Fig. S5). These findings suggest that microbially sourced 5-HTP may exhibit superior efficacy, possibly due to its specific ecological niche or probiotic properties of the strain itself. 5-HTP has not progressed to clinical use as a therapeutic agent, likely due to its rapid pharmacokinetics. The half-life of 5-HTP is just 2 h^95^. In mouse models, 5-HTP delivered as slow-release through chow improved its therapeutic properties than 5-HTP delivered as immediate-release by gavage^74^^,^^75^. Slow-release delivery can provide sustained drug exposure, lower *C*_max_, and significantly enhance the efficacy and/or safety of compounds with fast pharmacokinetics^96^. 5-HTP derived from gut microbiota acts more like a slow-release medication rather than an acute administration medication, which may explain the different effects of the EcN_5-HTP and the 5-HTP reagent. Although our study demonstrates EcN_5-HTP's behavioral effects while showing no significant changes in bulk brain serotonin (5-HT) levels, the whole-tissue measurement approach cannot exclude localized 5-HT production within specific neural circuits. This technical limitation leaves open the possibility that spatially restricted 5-HTP conversion in synaptic microdomains may have contributed to the observed behavioral outcomes.

Furthermore, transcriptomic analysis of the hippocampus in transgenic mice in this study revealed changes not only in the neuroactive ligand–receptor signaling and MAPK signaling pathway but also in other pathways potentially associated with autism, especially immune-related pathways such as the IL-17 signaling pathway, the TGF-beta signaling pathway, and the Toll-like receptor signaling (Fig. S6D). The IL17/IL17RA pathway plays important roles in neurodevelopmental disorders. Interleukin-17a-producing T helper cells in mothers led to cortical defects and ASD behaviors in offspring, which can be alleviated by therapeutic targeting of interleukin-17a during gestation^97^. Decreased level of TGF-*β* mRNAs was observed in the hippocampus and prefrontal cortex of the early-life immune activation (EIA) ASD mouse model^98^. It is also reported that Toll-like receptor signaling is associated with ASD behaviors like repetitive and restricted behaviors^99^. The role of these immune regulatory pathways in MeCP2-TG is still unclear, although a link between the MeCP2 gene and the activation of the axis TLR4 (Toll-like receptor 4)-IRAK1 (interleukin-1 receptor associated kinase 1)-NF-*κ*B p65 has been reported^100^. Our immunohistochemical analysis demonstrated significantly reduced Iba-1 immunoreactivity following EcN_5-HTP treatment (Fig. S8B and C), suggesting a direct modulatory effect on microglial activation states that may underlie these pathway alterations.

Our mechanistic investigations initially focused on hippocampal changes, given this region's established role in the behavioral phenotypes of MeCP2-TG mice^101^. While preliminary analyses of the prefrontal cortex and amygdala revealed no significant alterations in dendritic morphology (Fig. S8D), this negative finding itself provides important insights. The region-specific effects suggest 5-HTP may preferentially modulate synaptic plasticity in hippocampal circuits, potentially reflecting differential expression of serotonergic receptors or metabolic enzymes across brain regions. Electrophysiological analyses revealed that 5-HTP treatment selectively reduced aberrant *γ*-band (30–80 Hz) power in MeCP2-TG mice, suggesting improved neural network stability, which aligns with the observed molecular changes (Fig. S8E). Future studies investigating region-specific serotonergic signaling pathways may further elucidate how 5-HTP restores network dynamics, paving the way for precision interventions in synaptic dysfunction.

## Author contributions

Conceptualization: Zhi Liu. Data curation: Bei Li, Min Li. Formal analysis: Bei Li, Qianqian Yang, Min Li. Methodology: Bei Li, Min Li. Visualization: Bei Li, Min Li. Resources: Bei Li, Ruizhen Li, Shihan Li, Ziming Huang, Man Gu, Weirong Xu, Hongju Yang. Performed experiments: Bei Li, Qianqian Yang, Junqing He, Zhiqi Zhang, Xuchun Wan, Zihao Mo. Investigation: Bei Li, Qianqian Yang, Junqing He, Zhiqi Zhang, Xuchun Wan, Zihao Mo. Funding acquisition: Zhi Liu, Wei-Hua Chen, Hongju Yang, Hanning Huang, Bei Li. Writing-original draft: Bei Li, Qianqian Yang. Writing-review & editing: Zhi Liu, Wei-Hua Chen, Hongju Yang, Ziming Huang, Wei Li.

## Conflicts of interest

All authors declare that no competing interests are associated with this study.

## Appendix A Supporting information

The following is the Supporting Information to this article.
