# Supplementary material for: Gut microbiota-derived 5-HTP penetrates the host blood–brain barrier and ameliorates autism symptoms
Source: Acta Pharm Sin B. 2026 Jun 16;16(9):6168–84. doi: 10.1016/j.apsb.2026.06.025 (PMC13589926; doi:10.1016/j.apsb.2026.06.025)
Supplement: Supplementary file 1 [file mmc1.pdf]

Supporting Information for

Original article

Gut microbiota-derived 5-HTP penetrates the host blood–brain barrier and ameliorates autism symptoms

Bei Li[a,b,†], Qianqian Yang[b,†], Min Li[c,†], Junqing He[b], Zhiqi Zhang[b], Xuchun Wan[b], Zihao Mo[b], Ruizhen Li[d], Shihan Li[d], Weirong Xu[e], Man Gu[f], Hanning Huang[g], Wei Li[b,*], Ziming Huang[e,*], Hongju Yang[f,*], Weihua Chen[c,*], Zhi Liu[b,*]

[a]*Hubei Key Laboratory of Resource Utilization and Quality Control of Characteristic Crops, College of Life Science and Technology, Hubei Engineering University, Xiaogan 432000, China*

[b]*Key Laboratory of Molecular Biophysics of the Ministry of Education, Hubei Key Laboratory of Bioinformatics and Molecular Imaging, Department of Biotechnology, College of Life Science and Technology, Huazhong University of Science and Technology, Wuhan 430074, China*

[c]*Key Laboratory of Molecular Biophysics of the Ministry of Education, Hubei Key Laboratory of Bioinformatics and Molecular Imaging, Center for Artificial Intelligence Biology, Department of Bioinformatics and Systems Biology, College of Life Science and Technology, Huazhong University of Science and Technology, Wuhan 430074, China*

[d]*Department of Child Health, Maternal and Child Health Hospital of Hubei Province, Tongji Medical College, Huazhong University of Science and Technology, Wuhan 430070, China*

[e]*Wuhan Children's Hospital, Tongji Medical College, Huazhong University of Science and Technology, Wuhan 430010, China*

[f]*Division of geriatric Gastroenterology, The First Afliated Hospital of Kunming Medical University, Kunming 650032, China*

[g]*Gaozhou People's Hospital, Gaozhou 525200, China*

Received 9 August 2025; received in revised form 7 June 2026; accepted 7 June 2026

*Corresponding authors.

E-mail addresses: zhiliu@hust.edu.cn (Zhi Liu), weihuachen@hust.edu.cn (Weihua Chen), yanghongju@kmmu.edu.cn (Hongju Yang), huangziming@hbfy.com (Ziming Huang), hulwei_009@163.com (Wei Li)

[†]These authors made equal contributions to this work.

Figure S1

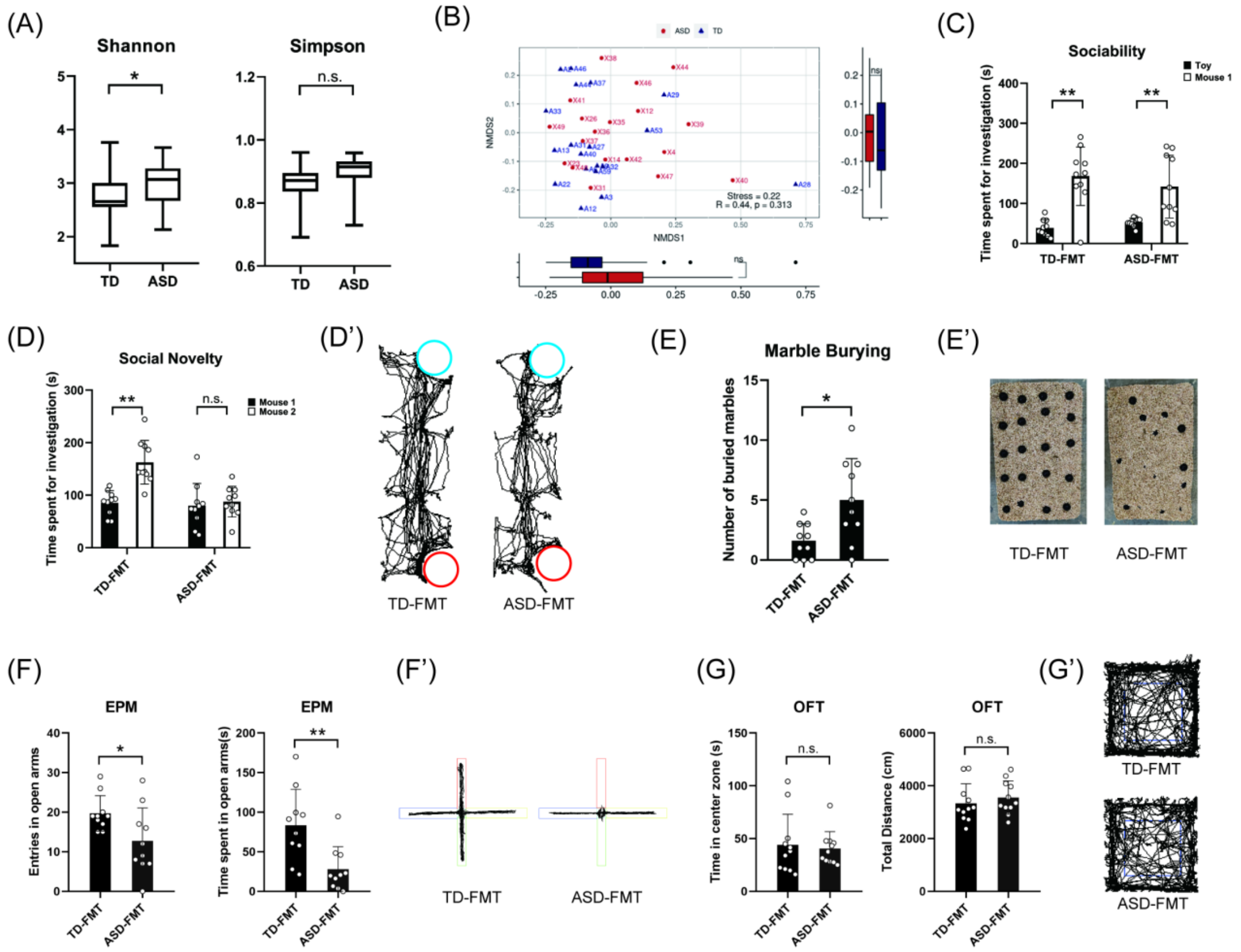

Figure S1 Mice colonized with dysbiotic gut microbiota from ASD children exhibit ASD-like behaviors. (A) α-diversity of fecal microbiota from typically developed children (TD, n = 20) and ASD (n = 20) children. (B) Principal coordinate analysis (PCoA) profile of microbial diversity in TD and ASD children. (C-D') 3-chamber sociability index. (E-E') Repetitive behavior by marble burying test. (F-F') Entries and time spent in open arms by elevated plus maze. (G-G') Entries and time spent by open field test. Mean values ± SDs are presented, p values were calculated using unpaired t-test, *p < 0.05 and **p < 0.01. n.s: not significant.

Figure S2

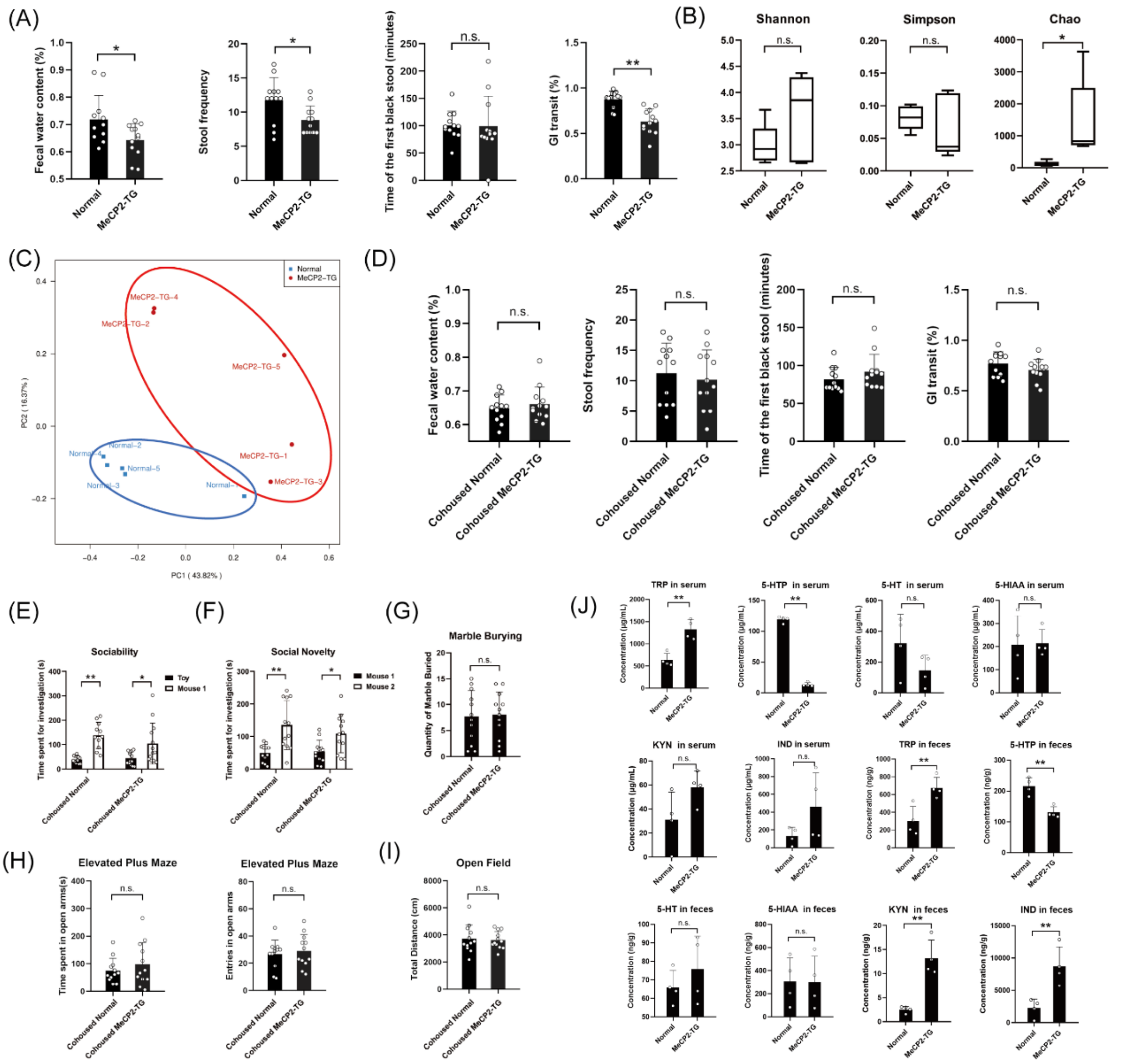

Figure S2 MeCP2-TG mice exhibit abnormalities in the intestine, distinct microbiome composition and reprogramed tryptophan metabolism. (A) GI function tests of Normal mice and MeCP2-TG mice. (B) α-diversity of fecal microbiota from normal mice and MeCP2-TG mice. (C) Principal coordinate analysis (PCoA) profile of microbial diversity from normal mice and MeCP2-TG mice. (D) GI function tests of co-housed normal mice and MeCP2-TG mice. (E-F) 3-chamber sociability index. (G) Repetitive behavior by marble burying test. (H) Total distance in by open field test. (J) Contents of tryptophan metabolites in serum and feces of normal mice and MeCP2-TG mice. Mean values ± SD are presented, p values were calculated using unpaired t-test, *p < 0.05 and **p < 0.01. n.s: not significant.

Figure S3

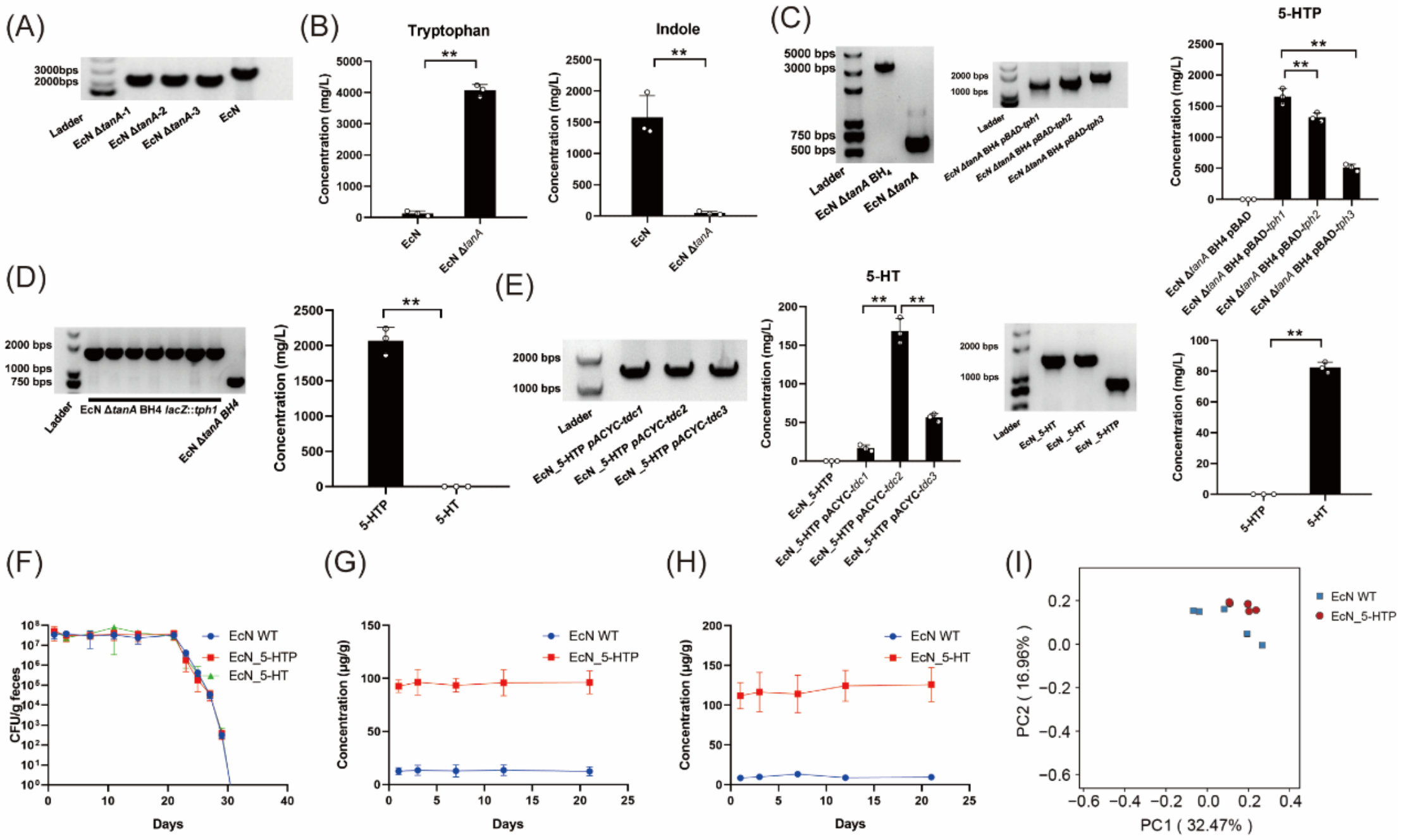

Figure S3 Engineering of EcN to synthesis of 5-HTP and 5-HT. (A) Gel electrophoresis image of *tanA* gene knockout. (B) EcN Δ*tanA* produced tryptophan and indole. (C) Gel electrophoresis image of BH_4 gene integrated into the genome of EcN Δ*tanA* and expression of different *tph* genes in EcN Δ*tanA* by plasmid. (D Gel electrophoresis image of *tph1* gene integrated into the genome of EcN Δ*tanA* BH_4 and production of 5-HTP and 5-HT. (E) Plasmid expression of different *tdc* genes and integration of *tdc2* into the genome of EcN 5-HTP. (F) Bacterial counts in feces samples collected at the indicated time intervals. (G-H) Contents of 5-HTP and 5-HT in fecal samples during the intervention period. (I) PCoA plot illustrating β-diversity differences in gut microbiota composition between EcN WT and EcN_5-HTP groups. Mean values ± SDs are presented, p values were calculated using unpaired t-test, $^{**}p < 0.01$.

Figure S4

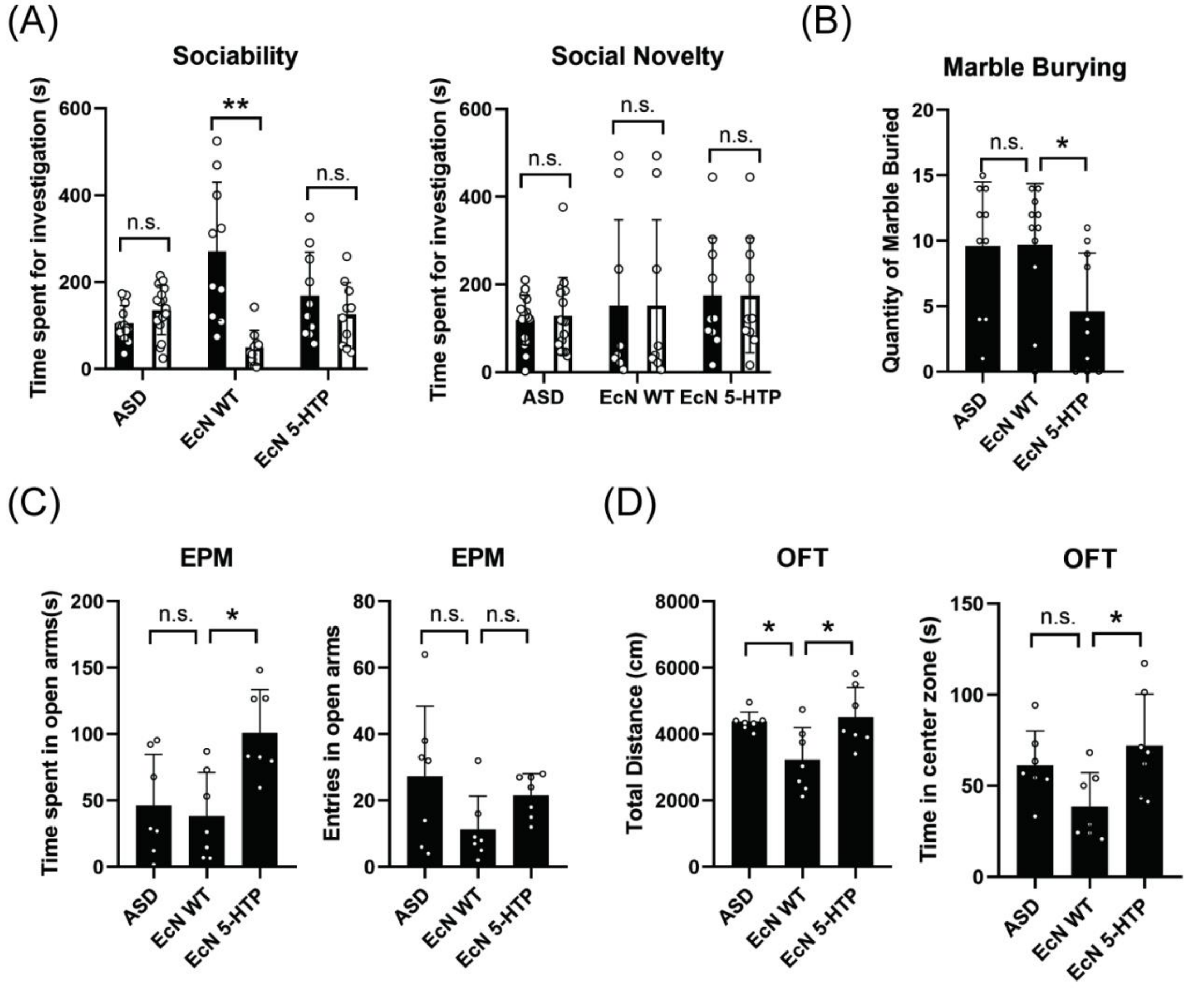

Figure S4 Microbiota-derived 5-HTP modulate behavioral abnormalities in BTBR mice. (A) 3-chamber sociability index. (B) Repetitive behavior by marble burying test. (C) Entries and time spent in open arms by elevated plus maze. (D) Total distance and time spent by open field test. Mean values ± SDs are presented, p values were calculated using unpaired t-test, $*p < 0.05$ and $**p < 0.01$. n.s: not significant.

Figure S5

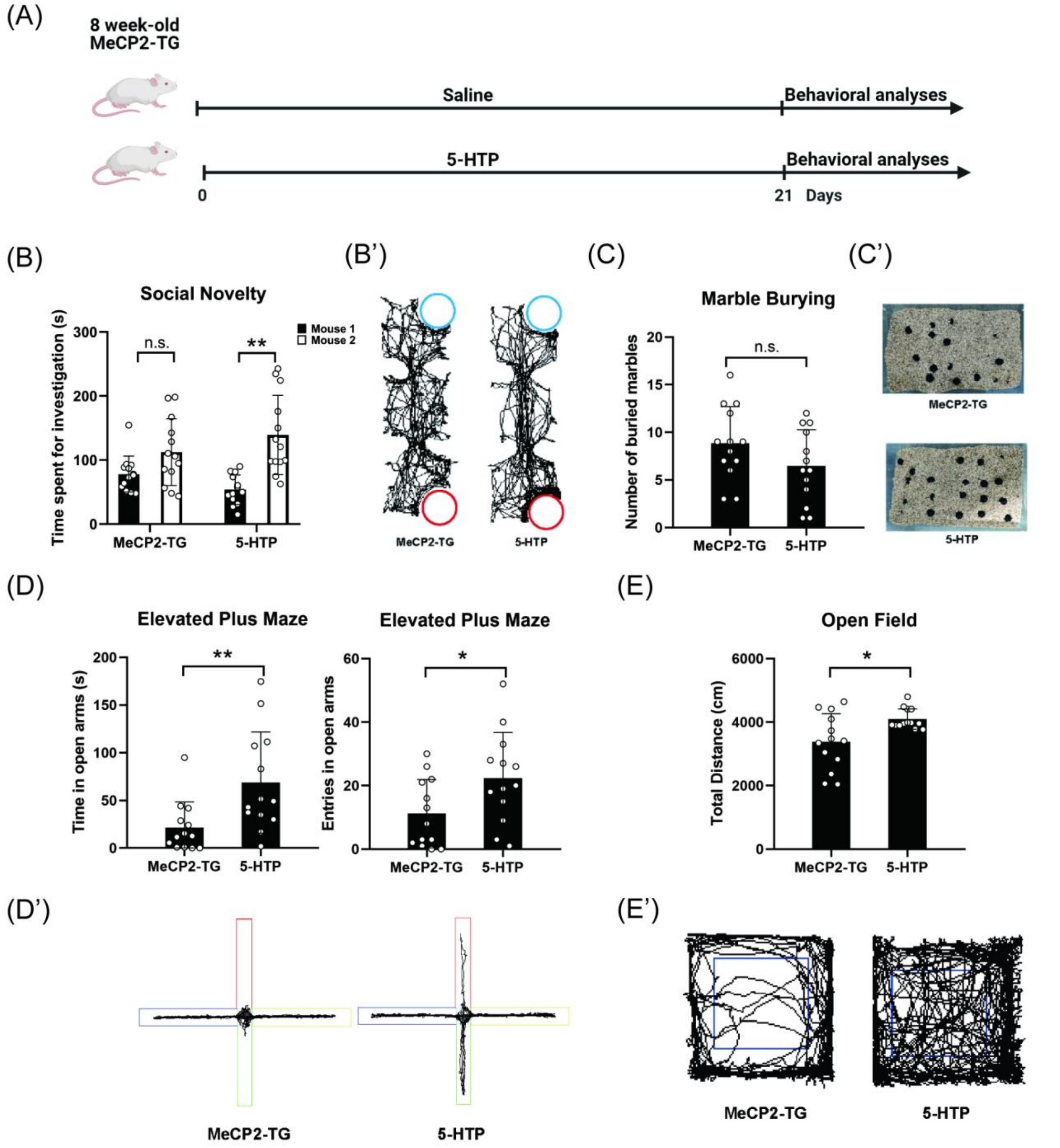

Figure S5 5-HTP modulate behavioral abnormalities in MeCP2-TG mice. (A) Experimental setup. (B-B') 3-chamber sociability index. (C=C') Repetitive behavior by marble burying test. (D-D') Entries and time spent in open arms by elevated plus maze. (E-E') Total distance by open field test. Mean values ± SDs are presented, p values were calculated using unpaired t-test, $*p < 0.05$ and $**p < 0.01$. n.s: not significant.

Figure S6

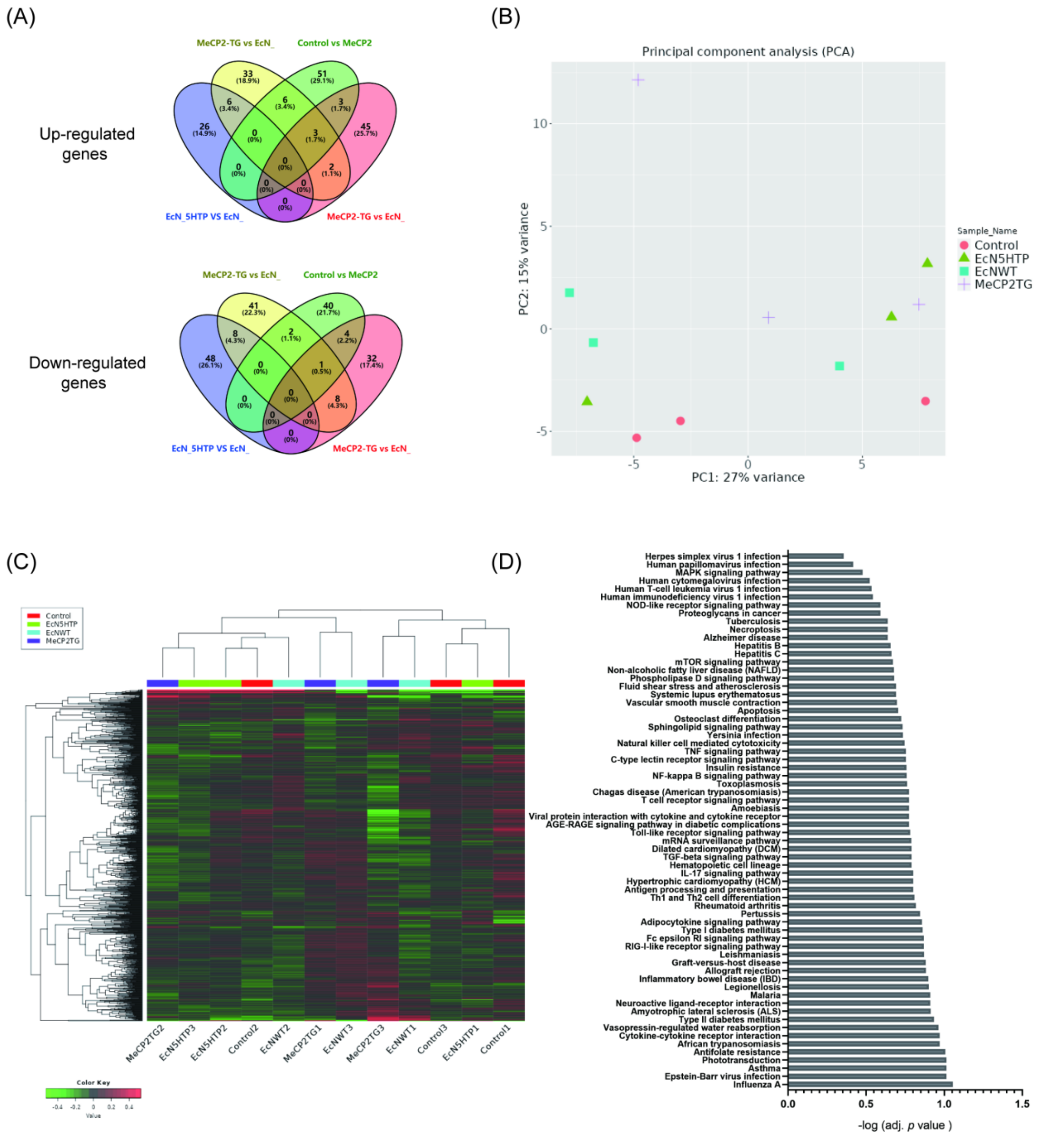

Figure S6 Analysis of RNA sequencing data of mice hippocampus after EcN 5-HTP treatment. (A) Petal plots illustrating the overlap of upregulated and downregulated genes between different groups. (B) Principal coordinate analysis (PCoA) profile of gene diversity. (C) Heatmap depicting differentially expressed genes of different samples. (D) Differential metabolic pathways between EcN WT treated group and EcN 5-HTP treated group.

Figure S7

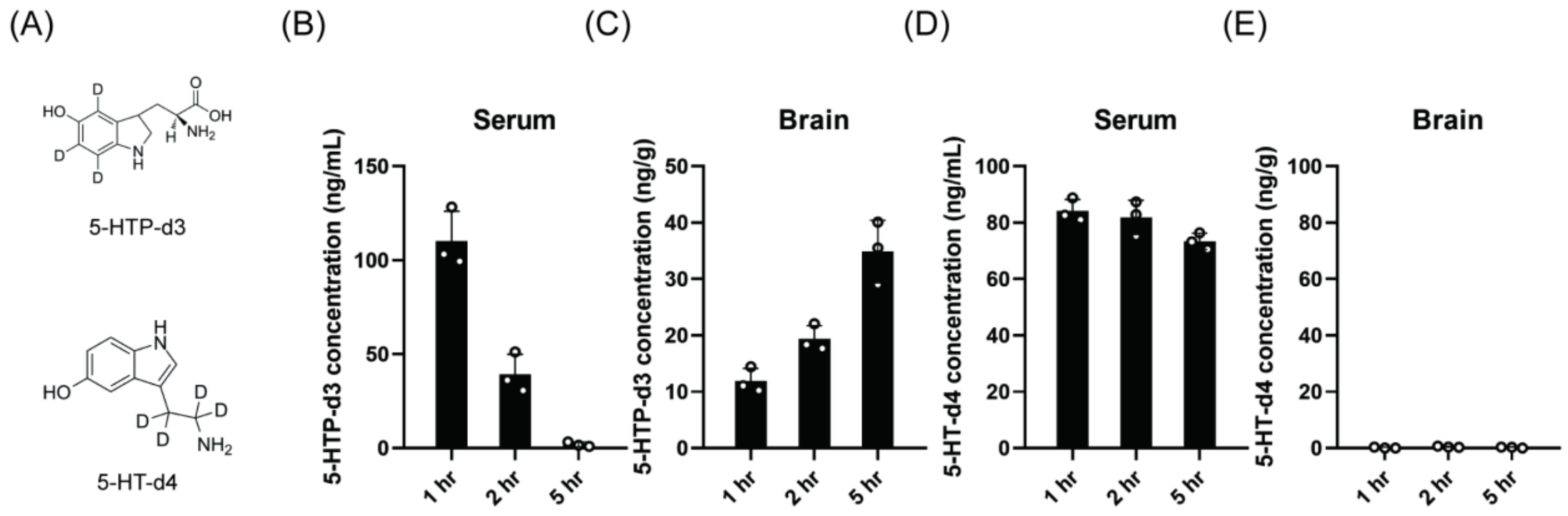

Figure S7 Microbiota-derived 5-HTP could cross the blood-brain barrier and affect host 5-HTP pool in normal mice. (A) 5-HTP and 5-HT labeled with deuterium. (B) Contents of 5-HTP-d3 in serum samples after gavage. (C) Contents of 5-HT-d4 in brain samples after gavage. (E) Contents of 5-HT-d4 in serum samples after gavage. (E) Contents of 5-HT-d4 in brain samples after gavage.

Figure S8

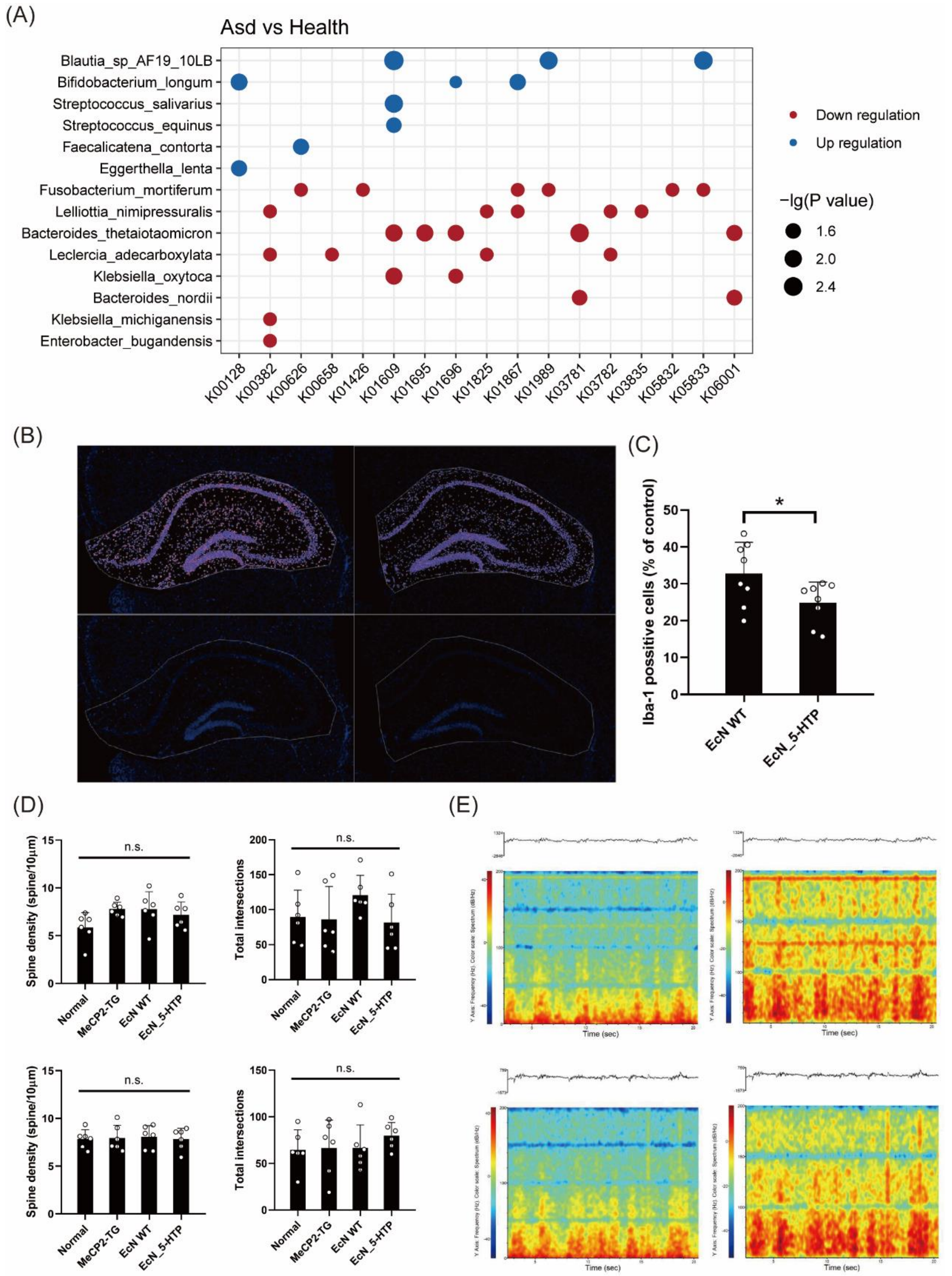

Figure S8 5-HTP modulate neuroinflammation and restoring neural network in MeCP2-TG mice. (A) Association network of microbial species and tryptophan metabolic enzymes in ASD patients of this study. Bubble plot illustrating significant associations between gut microbial species (Y-axis) and tryptophan metabolic enzymes (X-axis) in ASD versus healthy controls. Red bubbles indicate positive correlations, while blue bubbles show negative correlations. The circle size corresponds to the significance level, with larger circles denoting more statistically significant associations.

(B-C) The expression of Iba-1 in hippocampus after 5 weeks after treatment. Representable images of Iba-1/DAPI (red/blue) immunofluorescence staining (B) qualification of Iba-1staining in hippocampus region (C). (D) Dendritic spine density and arborization complexity in the prefrontal cortex (PFC, top) and amygdala (bottom) of MeCP2-TG mice following treatment. (B) (E) Representative local field potential (LFP) recordings and continuous wavelet transform spectrograms from MeCP2-TG mice (top) and EcN_5-HTP treated mice (bottom). Mean values ± SDs are presented, p values were calculated using unpaired t-test, $*p < 0.05$. n.s: not significant.

Table S1 Demographic and clinical characteristics of study participants with ASD.

ID	Age (y)	Sex	ABC	CARS	DSM-5(A)	DSM-5(B)	MRI	EEG	Metabolic Screen	Hearing Threshold (dB HL)	GI Symptoms
1	2.6	M	96	30	3	2	Normal	Normal	Normal	20	Constipation
2	2.8	M	64	31	3	2	Normal	Normal	Normal	15	Constipation
3	3.0	M	78	34	3	2	Normal	Normal	Normal	20	Constipation
4	3.3	M	98	30	3	2	Normal	Normal	Normal	20	Constipation
5	3.5	M	78	30	3	2	Normal	Normal	Normal	20	Diarrhea
6	3.5	M	73	30	3	2	Normal	Normal	Normal	20	Constipation
7	3.6	M	65	41	3	2	Normal	Normal	Normal	20	Constipation
8	3.7	M	47	35	3	2	Normal	Normal	Normal	20	Constipation
9	3.7	M	80	30	3	2	Normal	Normal	Normal	20	Constipation
10	4.0	M	66	27	3	2	Normal	Normal	Normal	20	Constipation
11	4.0	M	57	41	3	2	Normal	Normal	Normal	20	Constipation
12	4.0	M	56	34	3	2	Normal	Normal	Normal	20	Constipation
13	4.2	M	29	30	3	2	Normal	Normal	Normal	20	Constipation

14	4.6	M	60	35	3	2	Normal	Normal	Normal	25	Constipation
15	5.2	M	92	35	3	2	Normal	Normal	Normal	25	Constipation
16	2.0	F	84	33	3	2	Normal	Normal	Normal	10	Constipation
17	2.3	F	86	41	3	2	Normal	Normal	Normal	20	Diarrhea
18	3.2	F	75	30	3	2	Normal	Normal	Normal	10	Constipation
19	3.7	M	157	37	3	2	Normal	Normal	Normal	20	Constipation
20	4.0	F	90	35	3	2	Normal	Normal	Normal	10	Constipation

Table S2 MRM Transitions (Q1 f Q3), declustering potential (DP) and collision energy (CE) of the analytes.

Analyte	Q1 Mass (Da)	Q3 Mass (Da)	DP	CE
TRP	205.1	188.1	18	10
5-HTP	221.1	204.1	56	15
5-HT	177	160	23	13
5-HIAA	190	146	-87	-14
NAS	219.1	160	45	17
MET	233.1	174.1	61	21
IND	118.1	90.9	17	31
TRN	160.8	144.1	7	14
KYN	209	191.9	46	12

Table S3 Strain list.

Strains	Characteristics	Source
E. coli Nissle 1917 （EcN）	wildtype, *Str*^{R}	Lab collection
E. coli DH5α	Cloning host	Lab collection
EcN Δ*tnaA*	*tnaA* knockout, *Str*^{R}	This study
EcN WT	EcNΔ*tnaA*; *yicS/nep*::*BH*$_4$, *Str*^{R}	This study
EcN_5-HTP	EcNΔ*tnaA lacZ::p*$_{BAD}$*-tph1 yicS/nepS*::*BH*$_4$, *Str*^{R}	This study
EcN_5-HT	EcNΔ*tnaA lacZ::p*$_{BAD}$*-tph1*, *yicS/nep*::*BH*$_4$, *malEK::p*$_{BAD}$*-tdc*, *Str*^{R}	This study

Table S4 Plasmid list.

Plasmids	Characteristics	Source
pBAD24	Amp^R; Expression vector	Lab collection
pACYC-$_{ara}$BAD	Kan^R; Expression vector	Lab collection
pBAD-TPH1	pBAD24 with ORF of human *tph1*; Amp^R	This study
pBAD- TPH2	pBAD24 with ORF of human *tph2*; Amp^R	This study
pBAD-TPH3	pBAD24 with ORF of *Janthinobacterium lividum tph*; Amp^R	This study
pACYC-TDC1	pACYC with ORF of *Catharanthus roseus tdc*; Kan^R	This study
pACYC-TDC2	pACYC with ORF of *Oryza sativa* Japonica Group *tdc*; Kan^R	This study
pACYC-TDC3	pACYC with ORF of *Bacillus atrophaeus* strain C89 *tdc*; Kan^R	This study
pTargetF	repA101(Ts) P_{cas}-*cas9* P_{araB}-*Red* lacIq P_{trc}-sgRNA-*pMB1*; $Spec^R$	Lab collection
pCas	J23119(SpeI) promoter; temperature-sensitive; Kan^R	Lab collection
pKD46	P_{araB} promoter repA101, Amp^R	Lab collection
pKD3	Cm^R	Lab collection
pCP20	FLP, Rep101(Ts), temperature-sensitive; Amp^R	Lab collection

Table S5 Primer list.

Primers	Primer sequence (5'-3')
tnaA sgRNA-F	ATCACCAGTAACTCCGCAGGGTTTTAGAGCTAGAAATAGC
tnaA sgRNA-R	CCTGCGGAGTTACTGGTGATACTAGTATTATACCTAGGA
tnaA-1F	GAACTGCTGGCGGAAGAGTTG
tnaA-1R	GCCGCTGTCGGTCAGTAAATC
tnaA-2F	GATTTACTGACCGACAGCGGCGCTGAACTGCTGCGTTTAACC
tnaA-2R	TCAGCCCAAGGAACAATGAG
PTF sgRNA check-F	CAGTGAGCGAGGAAGCGGAAGAG
PTF sgRNA check-R	CCAGCAACGTCGGTTCGAGATGG
pCas check-F	CTTTGACAACGGCTCTATTCCC
pCas check-F	ATGGCTTTCTTCTGTTCACCTGA
EcN Δ*tnaA* check-F	GCGTGAGCATATCCACATTGAC
EcN Δ*tnaA* check-R	TCGTGCAATAGAGCCAGAAGAG
pBAD check-F	TAACAAAGCGGGACCAAAGC
pBAD check-R	GGCGTTTCACTTCTGAGTTC
pACYC check-F	GGGCATTAAACGAGTATCCC
pACYC check-R	CGCCTTATCCGGTAACTATC
yicS/nep sgRNA-F	GTCGGCAAAAGAGAAAACCAGTTTTAGAGCTAGAAATAGC
yicS/nep sgRNA-R	TGGTTTTCTCTTTTGCCGACACTAGTATTATACCTAGGA
yicS/nep-1F	CGAGTCGGTGCTTTTTTTGAATTCTCTAGGGATTATCGGTTATATCCAACGCGGTAG

yicS/nep-1R	GATCCCCTGCTGCCGGGGGCGACTCGGCCTG AGAAATGGCTG
BH_4-F	GGCCGAGTCGCCCCCGGCAGCAGGGGATCA TTTTGC
BH_4-R	GCAAATTTTACGACAGATAAAACGAAAGGC CCAGTC
yicS/nep-2F	CCTTTCGTTTTATCTGTCGTAAAATTTGCACG CCACAGGCGTC
yicS/nep-2R	GGGATAACAGGGTAATAGATCTAAGCTCCA GGTGAACCTTCGCATCAGAAAC
yicS/nep::BH_4 check-F	GACTATAGCGGTAAGGTC
yicS/nep::BH_4 check-R	AGCGTTGGTCAGTGGTTGTTG
lacZ sgRNA-F	GAAATTATCGATGAGCGTGTTTTAGAGCTAG AAATAGC
lacZ sgRNA-R	CCGCCACGCTCATCGATAATTTCACTAGTAT TATACCTAGGA
lacZ-1F	CGAGTCGGTGCTTTTTTTGAATTCTCTAGGTG CAGGAAACGCCAATAACATACAG
lacZ-1R	GGGATACTCGTTTAATGCCCATAACTATCCC GACCGCCTTACTGCAGCCTG
tph1-F	GGATAGTTATGGGCATTAAACGAGTATCCCG GCAG
tph1-R	CGTCGTTCCTACTCAGGAGAGCGTTCACCGA CAAAC
lacZ-2F	CGCTCTCCTGAGTAGGAACGACGTTGTAATA CGACGGCCAGAG
lacZ-2R	GGGATAACAGGGTAATAGATCTAAGCTGAT GGCGGAGCTGAATTACATTCC
lacZ::*tph1* check-F	AGATAAGCGCCCTGGAAACCGATG
lacZ::*tph1* check-R	CTAGTCCCGCTTTGAGCAACTG

PKD3 *malEK*-F	GACCTTTGAAGGTCGGCAGTACCGTTACACCATAATTCACTTTACTGGTGTGTAGGCTGGAGCTGCTTC
PKD3 *malEK*-R	CCATCTGGTGGCGGGTTTAACGTCCTCCTTAGTTCCTATTCC
tdc2-F	GGAATAGGAACTAAGGAGGACGTTAAACCCGCCACCAGATGG
tdc2-R	CGACGACCTGAACTTCACCCTCAAGGATGACGTCAGCGATATCACTCGGCTGCTGCAATGATACCGCGAGAC
malEK::*tdc2* check-F	TTCGGACTGGCGGCGTTAATAC
malEK::*tdc2* check-R	CTACCAACACCACGTCGTTCTG
